# Supplementary material for: Extraction of biological terms using large language models enhances the usability of metadata in the BioSample database
Source: Gigascience. 2025 Jun 23;14:giaf070. doi: 10.1093/gigascience/giaf070 (PMC12205978; doi:10.1093/gigascience/giaf070)
Supplement: giaf070_Supplemental_File [file giaf070_supplemental_file.docx]

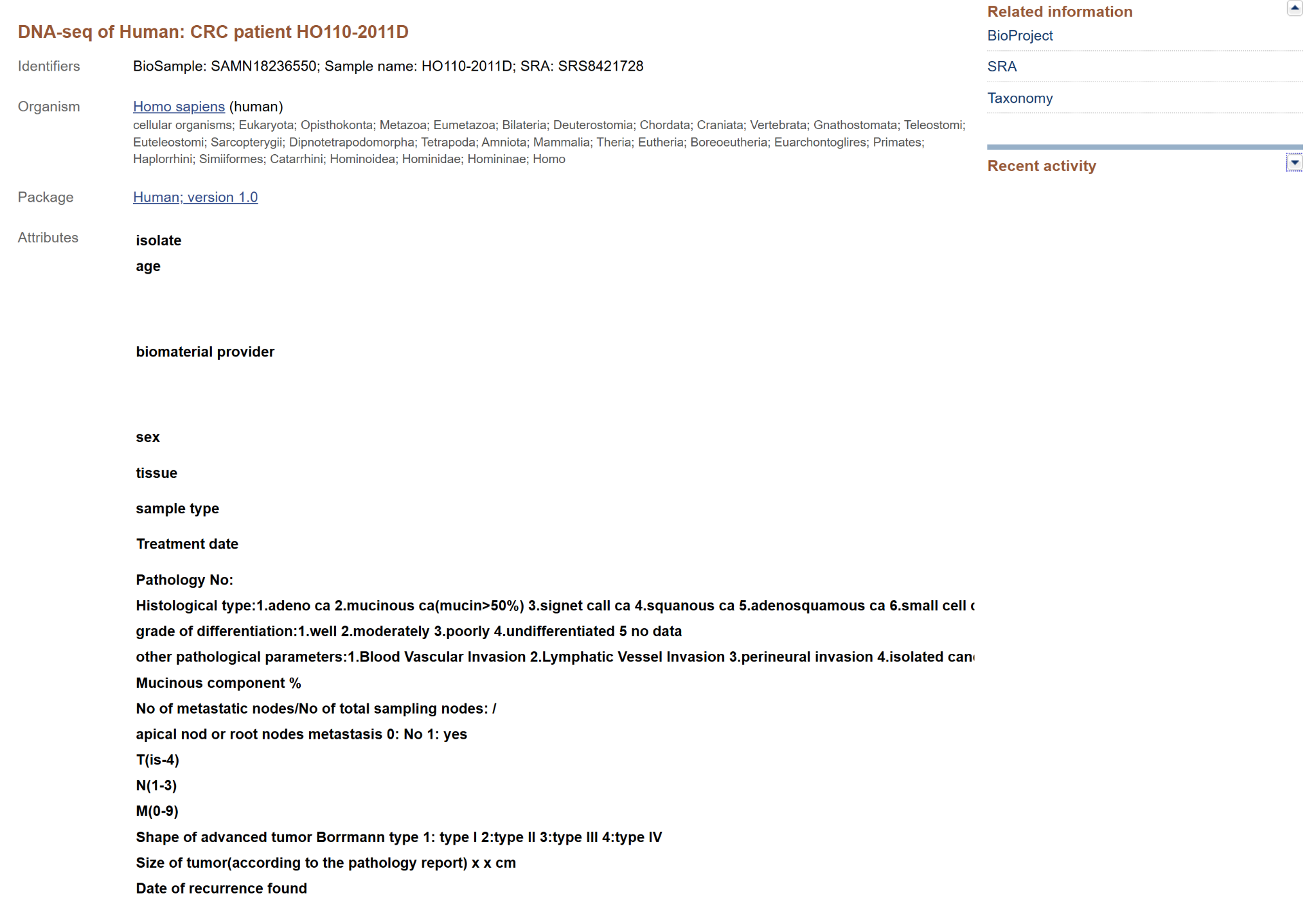


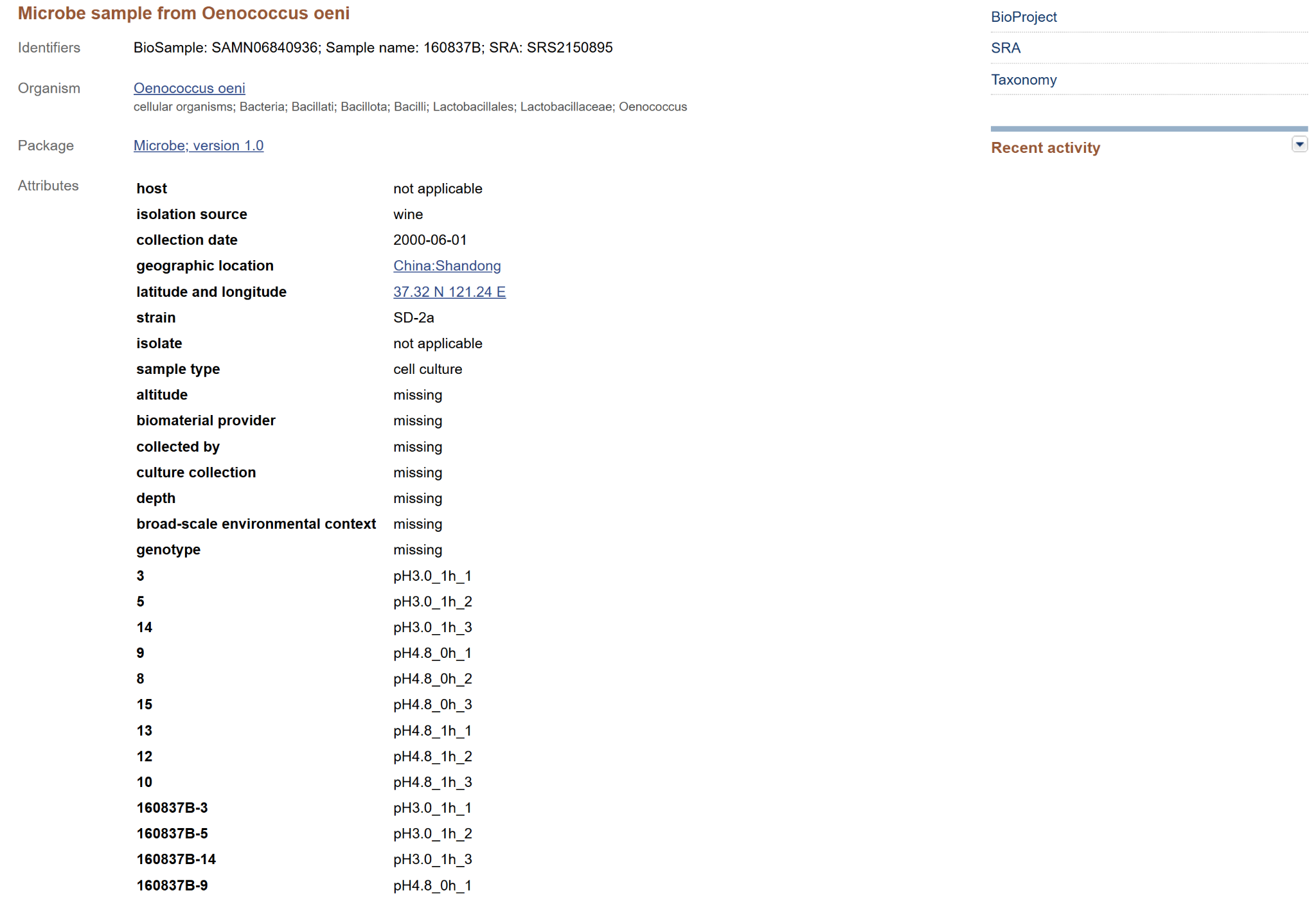


**Supplementary Figure 1.** Screenshots of NCBI BioSample webpages for records with attributes described in “less than ideal” ways. (A) SAMN18236550. This has attributes whose name defines option numbers and their corresponding values, resulting in an extreme length. Because the web interface of NCBI BioSample does not assume such long attribute names, it cannot display full-length attributes and their values. (B) SAMN06840936. This has uninterpretable attributes, such as “3” or “160837B-3.” Inferring from their values, they may mean pH and time for certain treatment, but we cannot say which condition was used for this specific sample.

**Supplementary Table 1.** Evaluation of the cell line extraction task per each experiment type by the conventional and proposed methods.

| **Experiment Type** | **Pipeline** | **Cell Line Accuracy** | **Cell Line Coverage** | **Non-cell line Precision** | **Non-cell Line Recall** |
| --- | --- | --- | --- | --- | --- |
| ATAC | MetaSRA | 0.910714 | 0.856383 | 0.784615 | 0.947059 |
| ChIP | MetaSRA | 0.896825 | 0.701149 | 0.672619 | 0.924242 |
| ATAC | LLM-assisted | 0.902985 | 0.957831 | 0.930769 | 0.935294 |
| ChIP | LLM-assisted | 0.939759 | 0.91791 | 0.928571 | 0.931818 |
