# Supplementary material for: Extraction of biological terms using large language models enhances the usability of metadata in the BioSample database
Source: Gigascience. 2025 Jun 23;14:giaf070. doi: 10.1093/gigascience/giaf070 (PMC12205978; doi:10.1093/gigascience/giaf070)
Supplement: giaf070_GIGA-D-25-00092_Revision_1 [file giaf070_giga-d-25-00092_revision_1.pdf]

GigaScience

Extraction of biological terms using large language models enhances the usability of metadata in the BioSample database

--Manuscript Draft--

**Manuscript Number:**	GIGA-D-25-00092R1
**Full Title:**	Extraction of biological terms using large language models enhances the usability of metadata in the BioSample database
**Article Type:**	Research
**Funding Information:**	JST NBDC — Not applicable<br>Japan Society for the Promotion of Science (24K20889) — Dr. Tazro Ohta<br>Research Organization of Information and Systems (045RP2023, 039RP2024) — Dr. Tazro Ohta
**Abstract:**	BioSample is a repository of experimental sample metadata. It is a comprehensive archive that enables searches of experiments, regardless of type. However, there is substantial variability in the submitted metadata due to the difficulty in defining comprehensive rules for describing it and the limited user awareness of best practices in creating it. This inconsistency poses considerable challenges to the findability and reusability of archived data.<br>Given the scale of BioSample, which hosts over 40 million records, manual curation is impractical. Automatic rule-based ontology mapping methods have been proposed to address this issue, but their effectiveness is limited by the heterogeneity of the metadata. Recently, large language models (LLMs) have gained attention in natural language processing and are promising tools for automating metadata curation. In this study, we evaluated the performance of LLMs in extracting cell line names from BioSample descriptions using a gold-standard dataset derived from ChIP-Atlas, a secondary database of epigenomics experiment data in which samples were manually curated. The LLM-assisted methods outperformed traditional approaches, achieving higher accuracy and coverage. We further extended them to extract information about experimentally manipulated genes from metadata when manual curation had not yet been applied in ChIP-Atlas. This also yielded successful results, including the facilitation of more precise filtering of the data and the prevention of possible misinterpretations caused by the inclusion of unintended data. These findings underscore the potential of LLMs in improving the findability and reusability of experimental data in general, which would considerably reduce the user workload and enable more effective scientific data management.
**Corresponding Author:**	Tazro Ohta<br>Chiba University Graduate School of Medicine School of Medicine: Chiba Daigaku Daigakuin Igaku Kenkyuin Igakubu<br>JAPAN
**Corresponding Author Secondary Information:**	
**Corresponding Author's Institution:**	Chiba University Graduate School of Medicine School of Medicine: Chiba Daigaku Daigakuin Igaku Kenkyuin Igakubu
**Corresponding Author's Secondary Institution:**	
**First Author:**	Shuya Ikeda, Ph. D.
**First Author Secondary Information:**	
**Order of Authors:**	Shuya Ikeda, Ph. D.<br>Zhaonan Zou, Ph. D.<br>Hidemasa Bono, Ph. D.

Powered by Editorial Manager® and ProduXion Manager® from Aries Systems Corporation

	Yuki Moriya, Ph. D.
	Shuichi Kawashima, Ph. D.
	Toshiaki Katayama, Ph. D.
	Shinya Oki, Ph. D.
	Tazro Ohta, Ph. D.
**Order of Authors Secondary Information:**	

Response to Reviewers:

We deeply thank the reviewers for their positive comments and suggestions. Our responses follow each reviewer contribution in the text below.

Responses to Reviewer #1

> 1. The gold-standard dataset constructed for evaluation, though carefully validated by experts, was limited to 600 samples (300 ChIP-seq and 300 ATAC-seq). Such a limited scope may introduce selection bias or fail to capture the full variability present across the entire BioSample database (>40 million records). It is unclear how representative these samples are of real-world metadata submissions. Clearly demonstrate the representativeness of the sample selection or increase sample size to better represent BioSample's diversity.

Response:
We agree with the reviewer's assessment of the natural limitation of sampling large populations. The BioSample database contains many idiosyncratic descriptions, such as those that have been provided by particular projects or data providers. Given the nature of the database, it would be challenging (if even possible) to select a dataset that could actually represent the entire BioSample database or even a large portion of it. In addition, we do not believe that increasing the size of the dataset used would dramatically change the conclusions of our study, despite the added resources and effort exerted. It is also nearly impossible to study the overall performance on the entire BioSample dataset, which is still growing.

ChIP-Atlas aggregates a comprehensive set of ChIP-seq and ATAC-seq datasets, and for our gold standard set, we randomly selected samples from different projects and cell types to mitigate potential bias. Based on the characteristics of the experimental procedure of these two sequencing techniques, it is reasonable to assume that the method's performance would be similar with a DNA/RNA sequencing dataset. However, metagenomic samples are more diverse, and our approach may not be sufficient to capture the characteristics of those types of samples. We do acknowledge that our dataset did not represent the full diversity of BioSample. Thus, we have explicitly added this information to the limitations portion of the Discussion section.

> 2. The manuscript predominantly compares the proposed LLM-based approach to the MetaSRA pipeline. While MetaSRA is a relevant baseline, the omission of comparisons with other contemporary methods like ChIP-GPT, and Bioformer is a notable oversight. These tools represent significant advancements in the field and have demonstrated efficacy in tasks closely related to the study's objectives. A comprehensive evaluation against these methods or comparative discussions would provide a clearer understanding of the proposed approach's relative performance and contributions.
https://academic.oup.com/bib/article/25/2/bbad535/7600389
https://pmc.ncbi.nlm.nih.gov/articles/PMC10029052/

Response:
We acknowledge this oversight regarding recent work, such as ChIP-GPT and Bioformer. ChIP-GPT has fine-tuned the LLaMA 1.0 model and incorporates prompt-engineering techniques, such as chain-of-thought prompting and pre-summarization steps, to perform named entity recognition (NER). Bioformer is a lightweight BERT-based model that can perform NER of biological concepts and is characterized by its compactness without sacrificing accuracy. We recognize that both are important recent works in regard to how machine-learning methods can provide solutions to the challenges of handling biological text and metadata.

Powered by Editorial Manager® and ProduXion Manager® from Aries Systems Corporation

We believe that our work would be better characterized as the next phase of the work illustrated by these examples rather than as being in competition with them. A key difference between our work and these studies lies in ontology mapping. Rather than merely extracting concepts, our approach links them to standardized ontologies, enabling an ontology-driven search that leverages their rich semantic information. We have updated the Introduction section to include a discussion of these prior works, and we have emphasized that our study directly addresses a research direction that these methods do not.

> 3. "LLM-assisted methods outperformed traditional approaches, achieving higher accuracy and coverage." While the study reports improved performance over MetaSRA, the absence of comparisons with other SOTA methods renders this assertion less robust. Without such comparative analyses, it's challenging to attribute the observed improvements solely to the proposed approach. Rephrasing claims to accurately reflect the scope of the comparisons made would strengthen clarity.

Response:
Further extending our response to the previous comment, to the best of our knowledge, no prior studies have performed ontology mapping on strings extracted using LLMs or similar methods. Therefore, we believe that comparing our method against algorithmic approaches, such as MetaSRA, is a valid and appropriate benchmark.

Moreover, one of the key contributions of our study is to demonstrate that recent general-purpose language models without domain-specific fine-tuning have made concept extraction considerably more accessible and accurate. We have revised the manuscript to clarify this point, emphasizing that the reported improvements highlight both an advancement in model capabilities and the novelty of incorporating ontology mapping.

> 4. Despite high accuracy, complex cases (fusion proteins, inhibitors mentioned indirectly, ambiguous terminology) were recognized as difficult, yet were excluded from primary accuracy evaluations. By excluding these ambiguous cases from performance metrics, the accuracy results might be artificially improved. Provide additional metrics that include these complex or ambiguous cases, clearly quantifying performance drops. This would offer more realistic insights into real-world applicability.

Response:
We excluded certain cases from the evaluation, not because of their complexity but because of the ambiguous nature of the problem. We found that the output of the LLM could be judged as correct or incorrect, depending on certain use cases. We did not anticipate these cases prior to our experiment; our prompt would have required a change to fairly evaluate performance with their inclusion. We have revised the manuscript to emphasize this point more clearly.

Given the context of the gene name extraction cases, which was a real-world application development trial, the primary purpose of reporting the accuracy was not to assert the superiority of our method but rather to provide a practical reference for how often incorrect outputs occurred under the given conditions and what developers and users could potentially expect. Including such context-sensitive cases in a single aggregated metric could misrepresent the true utility of the method, which is ultimately dependent on the user’s needs.

> 5. The error categorization provided (derivation issues, overlooked terms, selection failures, etc.) is helpful, but somewhat superficial. The deeper root causes—such as the LLM's lack of biological context knowledge, tokenization errors, or prompt ambiguity—were not thoroughly explored or explained. Discuss or perform deeper qualitative analysis on specific error instances, highlighting precisely why the LLM made incorrect decisions (e.g., lack of biological understanding, misinterpretation of abbreviations, limitations of prompt wording).

Response:
It is inherently difficult to identify precisely the root causes of LLM errors, as the decision-making processes of the models are black boxes. While certain tasks that were not possible with LLaMA 1 have become feasible with LLaMA 3, the only plausible inference from the user's standpoint is that the overall model performance has improved. We cannot determine whether a model truly possesses "biological context" knowledge, nor can we exhaustively explore all possible prompt variations, as prompt optimization is an open-ended process. Given these limitations, our focus was on whether the method performed sufficiently well for practical applications rather than on speculative analysis of the model internals.

> 6. Temperature settings were fixed at zero for deterministic outputs. While deterministic settings are valuable for reproducibility, exploring or reporting the effect of temperature variations on accuracy and robustness would have strengthened this methodological choice significantly.

Response:
We agree with the comment that it would be interesting to explore how temperature settings can affect overall performance. However, as stated in the manuscript, we aimed to develop a practical method that we could implement in real-world use cases to improve the usability of bioinformatics resources, such as ChIP-Atlas. In this context, as the reviewer agreed, temperature tuning would affect reproducibility, so we could not use it as part of the database development.

We believe that the reproducibility and transparency of LLM methods are factors that cannot be ignored by application developers in bioinformatics. Exploring the parameters and prompts that improve performance will likely not be a top priority for the time being, as the current nature of LLMs is such that their overall performance will improve as new models become available.

> 7. The authors have not sufficiently explored or justified their prompt engineering choices which are critical for reproducibility and optimization. I recommend providing additional experiments or discussions on alternative prompting strategies tested, including prompt variants that failed and reasons why particular prompts were selected.

Response:
We agree that exploring improvements through techniques such as chain-of-thought prompting, as done in ChIP-GPT, is certainly a viable approach. However, we observed that our simple prompt formulation had already achieved a performance comparable to that of ChIP-GPT. This suggests that performance improvements can be achieved through model refinement. We believe that we have demonstrated the practical value of the fact that general-purpose models perform very well with simple prompts, reducing the need for intensive prompt engineering. Within the context of the rapid pace of model improvement, we believe that the contribution of this research is the demonstration of strong performance with minimal prompt design.

Responses to Reviewer #2

> There are only a few minor issues in language usage and grammar that require attention. For example, there is a small typo in the description of gene overexpression ("achieved by trasfection of a plasmid…" on page 19) - "trasfection" should be "transfection" (unless this typo was carried over from the original prompt). Another example is the sentence "the outcomes of this study can handle these errors to rescue the affected published data for further use," which is a bit awkward in phrasing - perhaps reword to clarify that the methods developed can help correct metadata errors from submitted data.

Response:
We noticed this typographical error after the evaluation, but the model was robust

Powered by Editorial Manager® and ProduXion Manager® from Aries Systems Corporation

enough not to be affected by it. The results were still good; thus, we did not feel the need to perform the experiment again with a correction to the prompt. The prompt was reported accurately.

The manuscript was proofread before the initial submission and after our revision. We thank the reviewer for pointing out the text that still requires improvement. We have updated the sentence to clarify our original intent.

> Additional comments and suggestions: Beyond the points above, I have a few minor suggestions to further strengthen the manuscript. First, it would be helpful if the authors could clarify in the Methods how the manual evaluation of gene name extraction was performed—for example, whether multiple curators independently reviewed the outputs or if any consensus procedure was employed to resolve ambiguous cases. Providing this detail would add transparency to the accuracy figures reported, although the existing explanation about handling ambiguous cases (e.g., fusion genes) is already helpful.

Response:
We have added details to the Methods section describing the manual curation process for gene name extraction, as follows:
“An initial assessment was performed by one curator, followed by a review by a more experienced curator. For complex cases, the final judgment was made through discussion among four curators.”

> Second, given the manuscript's emphasis on a zero-shot LLM approach, it would be beneficial for the authors to briefly discuss whether alternative strategies, such as fine-tuning smaller language models, were considered. This would more clearly position the study within the broader landscape of metadata curation techniques.

Response:
Designing a fine-tuning dataset that adequately covers the diversity of BioSample metadata would be highly labor-intensive and difficult to use for generalization. From a practical standpoint, being able to achieve strong performance without fine-tuning—such as using zero-shot or few-shot prompting—is more desirable for real-world applications. Our results demonstrate that this is indeed feasible, and we have added a brief commentary on this point to the Discussion section to better position our approach within the broader landscape of metadata curation strategies.

> Third, the authors describe the use of the locally deployed Llama 3.1 model and emphasize its advantages regarding data privacy and scalability. Since these benefits are significant for practical adoption, it would further strengthen the manuscript if the authors explicitly highlight practical considerations, such as specific hardware requirements (in addition to the graphics card usage already included) and runtime performance benchmarks.

Response:
In the Methods section, we have added information about the required GPU memory size for running the Llama 3.1 model. Regarding runtime performance benchmarks, we considered this outside the scope of the current study, as there were no task-specific factors that would noticeably impact execution speed. However, we have already provided a general note on runtime performance in our Discussion section based on the specific tasks and computing environment used in the study.

> Finally, as mentioned earlier, the authors mention in Supplementary Table 1 that "no significant differences were observed" between ChIP-seq and ATAC-seq samples. If the term "significant" here is meant to indicate statistical significance, please include details of the specific statistical test and associated values (e.g., test statistics and p-values) that substantiate this conclusion. If no formal statistical testing was performed, it would be more appropriate to rephrase this statement to indicate a qualitative observation rather than imply statistical testing. These points are relatively minor and

	do not indicate fundamental issues with the manuscript.<br><br>Response:<br>As correctly pointed out by the reviewer, the statement in Supplementary Table 1 did not refer to statistical significance. We have rephrased the wording to clarify that this was a qualitative observation rather than the result of formal statistical inference testing.
**Additional Information:**	
**Question**	**Response**
Are you submitting this manuscript to a special series or article collection?	No
**Experimental design and statistics**<br><br>Full details of the experimental design and statistical methods used should be given in the Methods section, as detailed in our Minimum Standards Reporting Checklist. Information essential to interpreting the data presented should be made available in the figure legends.<br><br>Have you included all the information requested in your manuscript?	Yes
**Resources**<br><br>A description of all resources used, including antibodies, cell lines, animals and software tools, with enough information to allow them to be uniquely identified, should be included in the Methods section. Authors are strongly encouraged to cite Research Resource Identifiers (RRIDs) for antibodies, model organisms and tools, where possible.<br><br>Have you included the information requested as detailed in our Minimum Standards Reporting Checklist?	Yes
**Availability of data and materials**<br><br>All datasets and code on which the conclusions of the paper rely must be either included in your submission or deposited in publicly available repositories	Yes

(where available and ethically appropriate), referencing such data using a unique identifier in the references and in the “Availability of Data and Materials” section of your manuscript.<br><br>Have you have met the above requirement as detailed in our [Minimum Standards Reporting Checklist]?	
GigaScience has policies and guidelines in place for the use of generative AI-writing tools such as ChatGPT. If you have used such writing tools to assist with writing the manuscript this must be declared and cited in the text. Authors should not list AI-writing tools and other AI-assisted technologies as an author or co-author and should acknowledge that they are fully responsible for text generated or refined by AI-writing tools.<p><br><br>A summary of use (particularly in the introduction or among methods) needs to be included at the end of the paper, and the outputs should also be included as a supplementary file hosted in GigaDB or other open repositories. Please read our guidelines for more information. <p><br><br>By submitting to GigaScience, you are aware of the journal's AI-writing tools policy, and if you have declared use of such tools below, you have acknowledged this where appropriate in your manuscript and have made a summary of use and outputs available. <p><br><b>AI-assisted writing tools have been used in the preparation of this manuscript?	Yes

Manuscript Click here to access/download;Manuscript;manuscript.docx

Extraction of biological terms using large language models enhances the usability of metadata in the BioSample database

Shuya Ikeda[1,2], Zhaonan Zou[3], Hidemasa Bono[1,2,4], Yuki Moriya[1], Shuichi Kawashima[1], Toshiaki Katayama[1,5], Shinya Oki[3], Tazro Ohta[1,6,7†]

[1] Database Center for Life Science, Joint Support-Center for Data Science Research, Research Organization of Information and Systems

[2] Graduate School of Integrated Sciences for Life, Hiroshima University

[3] Institute of Resource Development and Analysis, Kumamoto University

[4] Genome Editing Innovation Center, Hiroshima University

[5] BioData Science Initiative, Joint Support-Center for Data Science Research, Research Organization of Information and Systems

[6] Department of Artificial Intelligence Medicine, Graduate School of Medicine, Chiba University

[7] Institute for Advanced Academic Research, Chiba University

[†] Corresponding Author

Authors' address, e-mail, and ORCID information:

- Shuya Ikeda
  - Univ. of Tokyo Kashiwanoha-campus Station Satellite 6F. 178-4-4 Wakashiba, Kashiwa-shi, Chiba 277-0871, JAPAN
  - sikeda@dbcls.rois.ac.jp
  - 0000-0002-1357-5159
- Zhaonan Zou
  - Gene Technology Center 6F. 2-2-1 Honjo, Chuo-ku, Kumamoto-shi, Kumamoto 860-0811, JAPAN

- zou@kumamoto-u.ac.jp
- 0000-0002-1075-4936

- Hidemasa Bono
  - Hiroshima University Innovation Plaza 3-10-23 Kagamiyama, Higashihiroshima-shi, Hiroshima 739-0046, JAPAN
  - bonohu@hiroshima-u.ac.jp
  - 0000-0003-4413-0651
- Yuki Moriya
  - Univ. of Tokyo Kashiwanoha-campus Station Satellite 6F. 178-4-4 Wakashiba, Kashiwa-shi, Chiba 277-0871, JAPAN
  - moriya@dbcls.rois.ac.jp
  - 0000-0001-8195-5893
- Shuichi Kawashima
  - Univ. of Tokyo Kashiwanoha-campus Station Satellite 6F. 178-4-4 Wakashiba, Kashiwa-shi, Chiba 277-0871, JAPAN
  - kwsm@dbcls.rois.ac.jp
  - 0000-0001-7883-3756

- Toshiaki Katayama
  - Univ. of Tokyo Kashiwanoha-campus Station Satellite 6F. 178-4-4 Wakashiba, Kashiwa-shi, Chiba 277-0871, JAPAN
  - ktym@dbcls.jp
  - 0000-0003-2391-0384
- Shinya Oki
  - Gene Technology Center 6F. 2-2-1 Honjo, Chuo-ku, Kumamoto-shi, Kumamoto 860-0811, JAPAN
  - okishinya@kumamoto-u.ac.jp
  - 0000-0002-4767-3259
- Tazro Ohta
  - 1-33 Yayoicho, Inage, Chiba, Chiba 263-8522, JAPAN
  - tazro.ohta@chiba-u.jp
  - 0000-0003-3777-5945

Abstract

BioSample is a repository of experimental sample metadata. It is a comprehensive archive that enables searches of experiments, regardless of type. However, there is substantial variability in the submitted metadata due to the difficulty in defining comprehensive rules for describing it and the limited user awareness of best practices in creating it. This inconsistency poses considerable challenges to the findability and reusability of archived data.

Given the scale of BioSample, which hosts over 40 million records, manual curation is impractical. Automatic rule-based ontology mapping methods have been proposed to address this issue, but their effectiveness is limited by the heterogeneity of the metadata. Recently, large language models (LLMs) have gained attention in natural language processing and are promising tools for automating metadata curation. In this study, we evaluated the performance of LLMs in extracting cell line names from BioSample descriptions using a gold-standard dataset derived from ChIP-Atlas, a secondary database of epigenomics experiment data in which samples were manually curated. The LLM-assisted methods outperformed traditional approaches, achieving higher accuracy and coverage. We further extended them to extract information about experimentally manipulated genes from metadata when manual curation had not yet been applied in ChIP-Atlas. This also yielded successful results, including the facilitation of more precise filtering of the data and the prevention of possible misinterpretations caused by the inclusion of unintended data. These findings underscore the potential of LLMs in improving the findability and reusability of experimental data in general, which would considerably reduce the user workload and enable more effective scientific data management.

Keywords

Automatic data curation, large language model, biological sample

Introduction

In recent years, advances in technologies, such as high-throughput sequencing for analyzing nucleotide sequences, have generated vast amounts of experimental data in the life sciences. To share and publish such experimental data, various public data repositories have been developed, such as the Sequence Read Archive (SRA) [1] for nucleotide sequence data and the Gene Expression Omnibus (GEO) [2] for gene expression analysis data. Since the creation of these repositories, a great number of experiments have been submitted to them and continue to be added at an increasing rate. As of November 2024, there were over 620,000 projects in SRA and 240,000 projects in GEO. The secondary analysis of accumulated public data in subsequent studies enhances the reliability of the experimental results and provides additional biological insights beyond those obtained by the original submitters. Moreover, secondary database services have been developed to collect public data on specific experiment types and to provide interfaces for browsing and analyzing such data. Examples include DEE2 [3] and GREIN [4] for gene expression analysis, and ChIP-Atlas [5] for epigenomics analysis, such as chromatin immunoprecipitation followed by sequencing (ChIP-seq).

Historically, sample metadata have been recorded in sample-specific records for each data repository. However, as more analyses have been conducted on the same samples, managing and searching for metadata within individual repositories has become increasingly cumbersome. To address this, the BioSample database was developed by the International Nucleotide Sequence Database Collaboration (INSDC), which is a joint effort among the National Center for Biotechnology Information (NCBI) in the United States, European Bioinformatics Institute (EBI), and DNA Databank of Japan (DDBJ), to centrally store sample information independent of the experimental type [6]. As of November 2024, BioSample hosts over 40 million records. Users seeking data of interest from these

repositories of massive amounts of data typically search based on experimental conditions and sample information. Information describing "data about experimental data" is referred to as metadata.

In BioSample, metadata, such as organism, tissue or cell type, disease, and treatment conditions, are described as key–value pairs (Fig. 1). The experimental conditions that can be described as metadata vary widely, making it difficult for database designers and administrators to define standardized rules to describe them. While packages specifying the required metadata for certain experiment types have been introduced, a generic package also exists with no predefined requirements. According to Gonçalves and Musen [7], 85% of BioSample records use the generic package. Consequently, much of the metadata description is left to the discretion of submitters, resulting in potential inconsistencies in the database, even for entries with identical experimental conditions. This situation undermines the purpose of public data repositories, which is to enable data reuse by other researchers.

Sample from Triticum aestivum

Identifiers	BioSample: SAMEA10378938; SRA: ERS8594256	
Organism	Triticum aestivum (bread wheat)<br>cellular organisms; Eukaryota; Viridiplantae; Streptophyta; Streptophytina; Embryophyta; Tracheophyta; Euphyllophyta; Spermatophyta; Magnoliopsida; Mesangiospermae; Liliopsida; Petrosaviidae; commelinids; Poales; Poaceae; BOP clade; Pooideae; Triticodae; Triticeae; Triticinae; Triticum	
Attributes	**collection date**	not collected
	**description**	For DNA extraction, ten seeds of one genotype were grown in the greenhouse and a single, approximately 10 cm leaf was harvested from a 10-days-old seedling.
	**sample name**	Achat_602
	**accession name**	Achat
	**annuality**	winter type
	**biological material altitude**	112 m
	**biological material geographic location**	Germany
	**biological material latitude**	51,816
	**biological material longitude**	11,283
	**biological material ploidy**	hexaploid
	**checklist**	BSDC00001
	**cultivar passport source**	wheatpedigree
	**gb2.0 subproject**	RenSeq
	**genus**	Triticum
	**material source geographic location**	Austria
	**panel**	Elite cultivar
	**plant anatomical entity**	leaf
	**plant structure development stage**	seedling
	**project**	Genbank 2.0
	**sample id**	GAT_LIMS:2035978, 2088313
	**species**	aestivum
BioProject	PRJEB48219 Genbank 2.0 - RenSeq<br>Retrieve all samples from this project	

Figure 1. An example of a BioSample record (https://www.ncbi.nlm.nih.gov/biosample/SAMEA10378938). The attributes of the sample are described as a set of pairs of attribute keys (e.g., "plant anatomical entity") and their corresponding values (e.g., "leaf").

One specific issue is the use of multiple representations for the same concept. For instance, synonyms (e.g., neuron vs. nerve cell), abbreviations and their full name equivalents (e.g., hESC vs. human embryonic stem cell), variations in capitalization, and typographical errors caused by human error are common. Thus, users of BioSample face difficulties retrieving all samples of possible interest because there is no unified terminology for describing concepts. To address this issue, ontologies that describe metadata in BioSample submissions can be helpful. Ontologies structure domain-specific concepts semantically by defining hierarchies and synonyms for terms. Each component of an ontology, called an "ontology term," standardizes the description of a concept. Examples of ontologies in the life sciences include

Uberon ("Uber-Anatomy Ontology") [8] for anatomical concepts, Cell Ontology [9] for cell types, and Disease Ontology [10] for human diseases. If BioSample metadata were described using ontology terms, it would alleviate the difficulty of retrieving samples with identical conditions. However, ontology usage in BioSample remains limited. For example, while BioSample packages define that the "disease" attribute should use Disease Ontology terms for human samples (https://www.ncbi.nlm.nih.gov/biosample/docs/attributes/), our investigation in November 2024 revealed that only 148,876 out of 595,177 human samples with a "disease" attribute used strings matching existing Disease Ontology labels.

Several strategies can be considered when mapping BioSample metadata to ontologies. Manual curation by experts is the most primitive approach and can achieve high accuracy, but it suffers from low scalability. In ChIP-Atlas, for example, metadata from epigenomics experiments are manually annotated by experts using a controlled vocabulary. However, this manual curation is limited to specific attributes, such as antigens and cell types, and expanding its scope would require additional effort. Therefore, automated curation systems are needed if scalability is prioritized over accuracy.

An example of an effort to automatically map BioSample metadata to ontologies is MetaSRA [11]. MetaSRA maps key–value pairs in BioSample records to concepts, such as tissue, cell type, cell line, disease, and developmental stage, using ontologies, such as Uberon, Cell Ontology, Cellosaurus [12], Disease Ontology, and Experimental Factor Ontology [13]. MetaSRA employs fuzzy string matching to query ontologies for terms and maps identified terms to metadata. However, this strategy struggles with homonyms and fails to distinguish terms used in a negative context. While MetaSRA applies rules to reduce misannotations—such as permitting mapping to cell line terms only for attributes named "cell line" or "cell type"—information may still be missed because cell line data are not always described under these specific attribute names.

Inappropriate mappings may persist despite these rules. For instance, when long text descriptions are provided in attribute values, some strings may not represent the sample itself. Figure 1 illustrates a wheat sample with an attribute named “plant anatomical entity” to indicate that this sample derives from a leaf. This record also has a “description” attribute to describe the sampling protocol in natural language. However, the presence of the words “seeds” and “leaf” within this description poses a challenge for rule-based ontology mapping. While a human reader can easily determine that this sample was collected from a leaf, it is difficult to algorithmically determine that the word “seeds” appears in a procedural explanation and that this sample is not a seed.

To address the challenges inherent in rule-based approaches, machine-learning-based methods have been considered. However, conventional machine-learning techniques have struggled to address the vast variety of description patterns in BioSample due to the difficulty of preparing a sufficiently comprehensive training dataset. For instance, Klie et al. [14] aimed to enhance metadata attributes in BioSample using a deep-learning-based approach. They tackled a named entity recognition (NER) task, extracting word sequences from longer texts, such as sample titles, that likely represented values for specified attributes. They used key–value pairs of BioSample as training data to develop a model to learn strings deemed plausible as values for given attributes. While their model achieved high accuracy in extracting strings, maintaining this level of accuracy required excluding monograms from the training set, which posed a limitation for extracting concepts represented by single-word terms. Furthermore, the approach relied on straightforward extraction and lacked the ability to differentiate between strings used in negative contexts or in other nuanced scenarios.

Recent advances in natural language processing, such as bidirectional encoder representations from transformers (BERT) and large language models (LLMs), have outperformed traditional methods across a wide range of tasks. While BioSample lacks consistent rules to describe

metadata, it is typically understandable to human users. Language models trained on modern technologies could potentially interpret such metadata and reorganize it appropriately in describing samples. The application of BERT and LLMs to NER and the organization of academic terminology is being actively researched. For instance, Fang et al. [15] developed a compact BERT model pre-trained on PubMed abstracts and PubMed Central full-text articles and demonstrated high performance of the model in NER tasks in the biomedical context. Dagdelen et al. [16] demonstrated the use of LLMs to extract specific information from materials science papers and structure it as Javascript Object Notation (JSON) objects. Sundaram et al. [17] reorganized BioSample metadata according to the attributes defined in existing metadata support tools. Cinquin [18] fine-tuned a LLaMA model, incorporating techniques such as prompt refinement via chain-of-thought and a preliminary summarization step, to perform NER for cell line information and ChIP targets from ChIP-seq samples. These studies highlight the potential of LLMs to improve metadata organization and searchability.

As previously discussed, LLMs are expected to be effective in addressing challenges in BioSample metadata, such as inconsistent attribute names and values, as well as the semantic interpretation of text strings, enabling high-accuracy concept extraction that has been previously difficult to automate. Applying this approach to concepts not covered by manual curation could enhance secondary databases built on BioSample data, improving the user experience through increased searchability. While prior works [15][17][18] have addressed similar challenges, the rapid advancement of LLMs makes it important to evaluate the performance of updated models. Furthermore, previous studies employing LLMs for NER did not perform ontology mapping. Mapping extracted terms to ontologies would enable searches based on well-organized and semantically reliable information.

In this study, we first examined the previously noted heterogeneity in BioSample descriptions from a different perspective and validated the feasibility of using LLMs for BioSample metadata curation. We then evaluated the effectiveness of the current LLMs in curating the BioSample metadata. To quantitatively assess ontology mapping, a gold standard dataset was constructed based on the manually curated results of ChIP-Atlas. The evaluation demonstrated that LLM-based methods outperformed traditional approaches. Furthermore, the application of LLMs to extract experimentally manipulated gene names from metadata was conducted and manually evaluated, showing that LLMs achieved sufficient accuracy to aid users in refining their searches, despite some limitations posed by the complexity of the BioSample descriptions.

Methods

Construction of a Gold-Standard Dataset for Cell Line Name Extraction

To quantitatively evaluate the extraction task performed by the LLM, we constructed a gold-standard dataset that defined ontology terms to represent BioSample records. For its creation, manual curation results from ChIP-Atlas, an integrated epigenomics database, were utilized. ChIP-Atlas comprehensively collects data of the following types: ChIP-seq, assay for transposase-accessible chromatin with sequencing (ATAC-seq), and bisulfite sequencing from SRA without any filtering. ChIP-Atlas manually maps information on the tissues and cell types of the sample origins to its own controlled vocabulary by applying the expertise of developmental biology specialists. While the curated results from ChIP-Atlas are not mapped to any ontology, leveraging this curated dataset was deemed a more efficient and reliable approach for determining ontology terms representing the BioSample records compared to building one from scratch.

The following considerations were taken into account when selecting samples: First, metadata for samples used in ChIP-seq experiments usually include the names of proteins targeted by ChIP. Protein names, which typically consist of alphanumeric combinations, bear similarities to cell line names; therefore, the presence of protein names in metadata could influence the difficulty of the cell line extraction task. In contrast, ATAC-seq experiments do not target specific proteins and do not suffer from this issue. Thus, we selected 300 samples each from the ChIP-seq and ATAC-seq experiments and enabled evaluation within each type of experiment. Second, to avoid skewing the task's difficulty, the 300 samples selected from each experiment type were ensured to come from distinct projects. Similarly, samples with identical terms mapped by ChIP-Atlas curation results were excluded. Third, we included only human samples because of the availability of Cellosaurus, an ontology that includes over 110,000 human cell lines and enables the precise definition of cell line terms representing BioSample records.

For the selected samples, corresponding terms from the Cellosaurus ontology were identified and defined as the gold standard.

Automated Annotation using LLM

Setup for LLM Execution

We employed Ollama [19] to run the Llama 3.1 70B instruct q4_0 model [20], which requires at least 35 GB of VRAM, on a machine equipped with an Nvidia RTX 6000 Ada GPU (48 GB of VRAM). To ensure the reproducibility of the results, the temperature parameter was set to 0. The source code for the task-specific prompts and input–output processing is publicly available on GitHub (https://github.com/sh-ikeda/bsllmner).

Cell Line Name Extraction Task

We designed a pipeline for performing the cell line name extraction task (Fig. 2). In this task, we used the set of attributes describing each BioSample record as input, prompting the LLM to extract the name of the cell line considered to represent the sample. The prompt (Prompt 1) provided a general definition of the cell lines, followed by instructions to analyze JSON-formatted data that contained key–value pairs of the sample attributes. The LLM was tasked with determining whether the sample was a cell line and, if so, extracting the cell line name. The extracted cell line names were used as the values for the “cell_line” attribute in the JSON files, which were then processed using MetaSRA to map them to ontology terms. We did not use LLMs for ontology mapping due to the frequently observed hallucination issues in which irrelevant ontology term IDs are presented, a problem inherent in LLMs. For samples resulting in multiple ontology terms with the same cell line name, further refinement was performed by the LLM. Each ontology term’s description was provided to the LLM, which compared this information with the BioSample metadata to output the most appropriate term (Prompt 2). The Cellosaurus information used in this process included the main label of the cell line (“name”), synonyms (“related_synonyms” and “exact_synonyms”), associated diseases (“diseases”), cell line type such as cancer cell line or embryonic stem cell line (“cell line type”), and the sex of the originating individual (“sex”). These details were appended to the end of Prompt 2 in JSON format, as shown below:

```
{
  "id": "CVCL:4719",
  "name": "S-2",
  "related_synonyms": [ "S 2",  "S2"],
  "exact_synonyms": ["s-2"],
  "diseases": ["Lung small cell carcinoma" ],
  "cell line type": ["Cancer_cell_line"],
  "sex": ["Male"]
}
```


To improve performance, the “think step by step” method was applied in the prompts. This method adds the phrase “think step by step” to the end of a prompt, encouraging the LLM to output not only the solution but also the reasoning process, a practice reported to enhance accuracy [21].

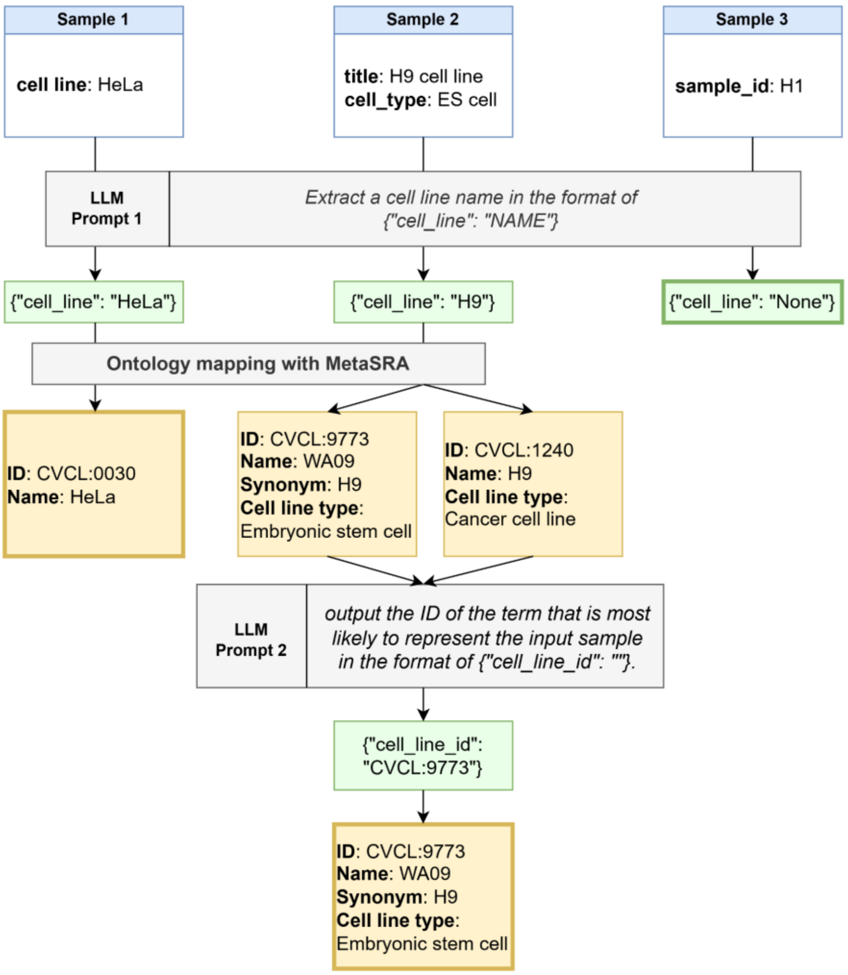

Figure 2. A flowchart describing the LLM-assisted ontology mapping pipeline of BioSample.

Ontology Mapping

We used a method based on MetaSRA for ontology mapping from extracted strings to Cellosaurus terms (https://github.com/sh-ikeda/MetaSRA-pipeline). Because the original MetaSRA pipeline (https://github.com/deweylab/MetaSRA-pipeline/) was implemented in Python 2, we ported it to Python 3 for better maintainability. Additionally, we made the following refinements:

- Improved the handling of cases in which ontology term labels or synonyms differed from the query only in capitalization, ensuring complete support for such scenarios.

- Replaced delimiter characters (such as “_” and “-”) in the input data with spaces and included the resulting strings as part of the query.

In the original implementation, strings with a length of 2 were excluded from the search to prevent mismapping, with a few exceptions. We modified this to allow all such strings without restriction because wrongly mapped terms were expected to be filtered in the selection phase by the LLMs (Prompt 2).

Evaluation Score Calculation

For the comparative evaluation of the existing method and our proposed LLM-assisted methods, we calculated the following metrics (Fig. 3):

- Accuracy and coverage for the task of mapping the correct cell line name to cell line samples
    - **Cell line accuracy** = (number of outputs mapping to a cell line that are correct) / (total number of outputs mapping to a cell line)
    - **Cell line coverage** = (number of correct cell line mappings included in the outputs) / (total number of gold standard entries for cell lines)
- Precision and recall for the task of identifying samples that are not cell lines
    - **Non-cell line precision** = (number of outputs not mapping to a cell line that are correct) / (total number of outputs not mapping to a cell line)
    - **Non-cell line recall** = (number of correct non-cell line entries included in the outputs) / (total number of gold standard entries for non-cell lines)

In cases where multiple cell line terms were suggested by the pipelines, we considered the output incorrect, as the correct cell line was not uniquely identified, even if the candidates included the correct cell line.

Class	Example	
	Gold Standard	Pipeline output
A: correct cell line	CVCL:1532 NCI-H2126	CVCL:1532 NCI-H2126
B: uncertain cell line	CVCL:0035 PC-3	CVCL:0035 PC-3, CVCL:4011 PaCa-3
B: wrong cell line	CVCL:0367 Jurkat E6.1	CVCL:0065 Jurkat
C: missed cell line	CVCL:M823 LPS141	(Not a cell line)
D: correct non-cell line	(Not a cell line)	(Not a cell line)
E: missed non-cell line	(Not a cell line)	CVCL:9773 H9

$$Cell\_line\_Accuracy = \frac{A}{A+B+E}$$

$$Cell\_line\_Coverage = \frac{A}{A+B+C}$$

$$Non\_Cell\_line\_Precision = \frac{D}{C+D}$$

$$Non\_Cell\_line\_Recall = \frac{D}{D+E}$$

Figure 3. Definition of the metrics used for the ontology mapping pipelines.

Gene Name Extraction Task

To extract gene names experimentally modulated in expression, we used human samples from the ATAC-seq and ChIP-seq projects registered in ChIP-Atlas. For the ATAC-seq samples, we randomly selected one sample from each of the 1,794 human projects registered in ChIP-Atlas. For ChIP-seq samples, because the number of registered projects was relatively large (4,806), we first randomly selected 2,000 projects as a subset and then randomly chose one sample from each. We used the EBI BioSamples API to obtain the sample data, but data could not be obtained for 42 ATAC-seq samples and 29 ChIP-seq samples. As a result, we used 1,752 ATAC-seq samples and 1,971 ChIP-seq samples for the task.

The prompt (Prompt 3) instructed the LLM to analyze JSON-formatted BioSample metadata and output a list of modulated genes and their respective modulation methods as a JSON array. Because a single sample might involve the modulation of multiple genes, the output array consisted of JSON objects with “gene” and “method” attributes, such as the following:

[{"gene": "ARID1A", "method": "knockout"}, {"gene": "CHAF1A", "method": "dTAG"}]

The prompt began by defining the major modulation methods: gene knockout, knockdown, and overexpression. These are prone to considerable variability in terminology, as shown in the following examples:

- **Knockout** can appear as “knockout,” “KO,” “-/-,” or “deletion.”
- **Knockdown** may be described using “knockdown,” “KD,” “shRNA,” “siRNA,” “si(Target gene name),” or “RNAi.”
- **Overexpression** might include terms such as “overexpression,” “OE,” “transfection,” or “transduction.”

If a gene was identified in the metadata as modulated by one of these methods, the output’s “method” attribute was aligned to these standard terms. For other methods described in the input, the prompt instructed the LLM to extract and retain the method name, as is. During the experiment, a typographical error was missed in this prompt. “Trasfection” was inadvertently used instead of the correctly written “transfection.” This was not noticed until after the evaluation. However, the model was robust and did not appear to be adversely affected by it. It is reported here with the error intact in the interest of transparency and accuracy. The extracted results were manually evaluated. An initial assessment was performed by one curator, followed by a review by a more experienced curator. For complex cases, the final judgment was made through discussion among four curators. Samples with descriptions that made it difficult to yield a definitive result under the current prompts were excluded from the evaluation (examples are provided in later sections).

Prompt 1. Cell line extraction

A cell line is a group of cells that are genetically identical and have been cultured in a laboratory setting. For example, HeLa, Jurkat, HEK293, etc. are names of commonly used cell lines.

I will input json formatted metadata of a sample for a biological experiment. If the sample is considered to be a cell line, extract the cell line name from the input data.

Your output must be JSON format, like {"cell_line": "NAME"} . "NAME" is just a place holder. Replace this with a string you extract.

When input sample data is not of a cell line, you are not supposed to extract any text from input. If you can not find a cell line name in input, your output is like {"cell_line": "None"} . Are you ready?

Prompt 2. Cell line selection

I searched an ontology for the cell line, "{{cell_line}}". I have found multiple terms which may represent the sample. Below are the annotations for each term. For each term, compare it with the input JSON of the sample and show your confidence score (a value between 0-1) about to what extent the entry represents the sample. In the comparison, consider the information such as:

Whether the term has a name or a synonym exactly matches the extracted cell line name, "{{cell_line}}".

Whether the term has disease or cell line type information which matches sample information.

Based on the confidence score, output the ID of the term that is most likely to represent the input sample in the format of {"cell_line_id": ""}. If it is not clear which one is most likely from the given information, output {"cell_line_id": "not unique"}.

Prompt 3. Gene name and gene modulation method extraction

There are several experimental methods to modulate gene expression.

Gene knockout (KO), also known as gene deletion, involves completely eliminating the expression of a target gene by replacing it with a non-functional version, usually through homologous recombination in cells or animals. This results in a complete loss of the gene's function.

Meanwhile, gene knockdown (KD), also known as RNA interference (RNAi), involves reducing the expression of a target gene without completely eliminating it. KD is achieved by introducing small RNA molecules, siRNA or shRNA, that specifically bind to and degrade the messenger RNA (mRNA) of the target gene.

Gene overexpression refers to the process of increasing the expression of a specific gene beyond its normal levels in a cell. This is achieved by trasfection of a plasmid carrying the gene of interest, transduction of viruses carrying the gene of interest, etc.

I will input json formatted metadata of a sample for a biological experiment. If the sample is considered to have genes whose expression is experimentally modulated, extract the gene names from the input data and specify the modulation method.

Your output must be in JSON format, like [{"gene": "GENE_NAME", "method": "METHOD_NAME"}] . "GENE_NAME" and "METHOD_NAME" are placeholders. Replace them with the gene name you extract and the modulation method name you specify, respectively. If the modulation method is either gene knockout, gene knockdown, or gene overexpression, the value of the "method" attribute must be "knockout", "knockdown", and "overexpression", respectively. Otherwise, the value of the "method" attribute must be the method name found in the input data.

If the input sample data is not considered to have genes whose expression is modulated, your output JSON must be an empty list (namely, '[]'). Note that multiple genes can be modulated in one sample. In this case, be sure to include all of them in the list of the output JSON. For example, if you find "PRNP" and "MSTN" as knocked out genes, your output must be [{"gene": "PRNP", "method": "knockout"}, {"gene": "MSTN", "method": "knockout"}]. Note also that multiple gene modulation methods can be used for one sample. For example, you may find "ARID1A" as a knocked-out gene and "CHAF1A" as a gene treated with dTAG. In this case, your output must be [{"gene": "ARID1A", "method": "knockout"}, {"gene": "CHAF1A", "method": "dTAG"}].

Are you ready?

Mapping Gene Names to Gene IDs

To map gene names extracted using the LLM to gene IDs, we utilized the HUGO Gene Nomenclature Committee (HGNC) multi-symbol checker (https://www.genenames.org/tools/multi-symbol-checker/). This tool allows for searching input strings and retrieving corresponding IDs, including not only approved official symbols but also previous, alias, and withdrawn symbols. We included all these options for mapping and set the “Search case” parameter to “insensitive” because gene names in the BioSample metadata were not always the current official symbols.

Results

Survey of the BioSample Metadata

As highlighted by Gonçalves and Musen [7], BioSample attribute names exhibit considerable variability, complicating rule-based automated metadata organization. To confirm this, we conducted investigations into several aspects of BioSample metadata variability.

To assess the variability in attribute names used to represent cell lines, we examined the attribute names associated with the value “HEK293T,” a commonly used and distinctive cell line name. It is unlikely that “HEK293T” would be used to refer to anything other than this cell line. Despite this specificity, 27 different attribute names were found to be used for this individual cell line (Table 1), demonstrating remarkable inconsistency, even for what could reasonably be considered a readily identifiable concept.

Table 1. Variability in the names of attributes whose value was "HEK293T"

Attribute Name	# of Projects	# of Samples
cell line	667	11,547
cell_line	435	96,516
source_name	338	4,972
isolate	88	2,641
cell type	33	326
strain	20	1,104
cell_type	7	507
tissue	6	442
spike-in cell line	6	385
cell line background	6	68
biomaterial_provider	4	102
human cell line	4	23
cell_subtype	3	952
lab_host	3	76
cell line/type	3	14
spike-in cell_line	2	68
cell-type	2	29
host cell line	2	8
cell lines	1	96
mix1	1	56
cell line/tissue	1	49
cell line/strain	1	26
lab host	1	10
cell line or tissue	1	6
dev_stage	1	4
isolation_source	1	2
cell	1	1

We also analyzed the frequency of attribute names associated with values containing the string "H1" (Table 2). "H1" is the name of a commonly used embryonic stem cell line, but this simple string can represent many different concepts other than a cell line. In fact, the attribute names containing "H1" values displayed substantially more variability, as shown in Table 2, and included names such as "well" and "genotype," which are unlikely to represent

cell lines. This highlights the greater ambiguity inherent in interpreting such simple strings in metadata.

Table 2. Variability of names of attributes whose value included “H1”

Attribute Name	# of Projects	# of Samples
cell line	299	4,734
source_name	234	4,360
cell type	74	1,043
cell_line	44	635
isolate	32	192
well	17	263
tissue	13	77
treatment	10	54
strain	9	160
genotype	8	59
cell_type	8	99
cell line/strain	8	51
sample type	5	30
sample name	5	25
individual	5	29
Submitter Id	5	5
submitted subject id	3	18
subject	3	128
cellline	3	52
subject id	2	8
sample_patient	2	2
line	2	95
genotype/variation	2	6
es cell line	2	32
donorid	2	187
donor id	2	19
donor cell line	2	116
description	2	14
condition	2	28
cell description	2	9
antibody targetdescription	2	2
LINE	2	95
well position	1	2
well id	1	1
uniqueid	1	1
treatment/time period	1	3
tissue-type	1	1

time	1	12
submitted sample id	1	1
subclone	1	10
stimulus	1	2
state	1	2
source cell line	1	4
source	1	2
short_name	1	1
shRNA	1	2
seq_id	1	2
sample_type	1	3
sample name in supplementary file	1	1
sample description	1	2
replicate	1	8
psc line	1	3
position in smart-seq2	1	14
position in library	1	11
plate_location	1	5
plate-position	1	4
phenotype	1	1
patient_id	1	1
patient id	1	4
parental cell line	1	8
other sample name	1	1
originating cell line	1	18
original_Sample_ID	1	1
origen of cultured cells	1	4
name in the manuscript	1	24
library	1	3
label	1	2
input	1	44
halplogroup	1	4
growth condition	1	3
grna	1	6
genetic perturbation	1	37
expression	1	3
donor samples	1	1
donor	1	379
differentiated from	1	5
depletion	1	2

crispr library	1	22
column in countmatrix	1	1
clone_id	1	1
cell source	1	8
cell lines	1	576
cell line of origin	1	20
cell line name	1	18
cell line background	1	6
biospecimen repository sample id	1	1
assay name	1	3
antibody	1	9
Sample Name	1	25
Name	1	1
DONOR_ID	1	1
DIFFERENTIATION_METHOD	1	26
ArrayExpress-StrainOrLine	1	9

We surveyed the usage frequency of all the attribute names. Because samples from the same project often share the same attributes, and the number of samples per project was highly skewed [11], we conducted the count on a project basis. As of June 7, 2024, BioSample contained 27,639,806 records associated with BioProject [6], featuring 76,282 unique attribute names. Of these, 57,750 (75.7%) attribute names were used in only a single project, and 73,207 names (96.0%) were used in 10 or fewer projects.

Examining individual records revealed instances in which data submitters seemed unfamiliar with proper metadata annotation practices. Some records contained incomprehensible strings or poorly described attributes, as shown in Table 3 and Supplementary Fig. 1.

Table 3. Examples of sample attributes published in “less than ideal” ways

BioSample ID	Attribute name	Attribute value
SAMEA2784244	scientific_name	root
SAMN09935219	{	url: "https://api-ui.mg-rast.org/metagenome/mgm4738303.3?verbosity=metadata",
SAMEA2024666	TODO: TAG NAME	TODO: TAG VALUE
SAMN06840936	3	pH3.0_1h_1
SAMD00009749	ACAGACAGCGT	CO2-day-56-F9-replicate2
SAMN18236550	Histological type:1.adeno ca 2.mucinous ca(mucin>50%) 3.signet call ca 4.squanous ca 5.adenosquamous ca 6.small cell ca 7.undifferentiated ca 8.carcinoma NOS 9.carcinoid 10.leiomyosarcoma 11.lymphoma 12.adenocarcinoid 13 others	not applicable
SAMN12753670	sample_name,sample_title,bioproject_accession,organism,host,isolation_source,collection_date,geo_loc_name,lat_lon,ref_biomaterial,rel_to_oxygen,samp_collect_device,samp_mat_process,samp_size,source_material_id,description	PI3,Phosphorus Inefficient,,metagenome,Apple,,13-Oct-18,China: Beijing,39.54 N 116.25 E,,,,,,PI biological replicate 3
SAMEA6935312	6784d92c744ac5dcc47f11a04c34e48e	90e6b91bb57ad7d13592b3fe79ab5ce0
SAMN35358032	filename	1123_BB_lib2_rep1_S49_R1_001.fastq.gz
SAMN15196600	description;;;;;;;;;;;;;;;;;;;;;;;;;;;;;;;	Tomato leaf microbiome;;;;;;;;;;;;;;;;;;;;;;;;;;;;;;;
SAMN27601919	Description	>hCoV-19/Mexico/CPALB32021033/2020 CGAAAGTTGGTTGGTTTGTTACCTGGG...[This continued in the original for about 30000 characters]
SAMN00760728	Description	#SampleID BarcodeSequence LinkerPrimerSequence Sites Description #These 8 samples are from Dianchi #Sediment sample 16S.1 ATGCTACGTC TTACCGCGGCTGCTGGCAC Caohai Caohai_Jun. 16S.2 ATGTGACTAC TTACCGCGGCTGCTGGCAC Waihai waihai_Jun. 16S.3 CACGAGACAG TTACCGCGGCTGCTGGCAC Caohai Caohai_Sep. 16S.4 CACGCGAGTC TTACCGCGGCTGCTGGCAC Waihai Waihai_Sep. 16S.5 CACGCTACGA TTACCGCGGCTGCTGGCAC Caohai Caohai_Dec. 16S.6 CACGTGTATA TTACCGCGGCTGCTGGCAC Waihai Waihai_Dec. 16S.7 CACTACGATG TTACCGCGGCTGCTGGCAC Caohai Caohai_Mar. 16S.8 CACTATACTC TTACCGCGGCTGCTGGCAC Waihai Waihai_Mar.

The extensive variability in attribute names and their usage is challenging for rule-based methods that attempt to ensure the comprehensive extraction of necessary information while avoiding erroneous interpretation from irrelevant data. This underscores the value of using LLMs for metadata organization, as they are able to flexibly interpret texts based on their contexts, even in a case like this with such complex and inconsistent descriptions.

Creation of the Gold Standard Dataset for Cell Line Extraction

To evaluate the performance of the cell line extraction task, we created a gold standard dataset (https://doi.org/10.5281/zenodo.14881142). This dataset included 300 samples derived from ChIP-seq experiments and 300 samples derived from ATAC-seq experiments. To ensure fairness in the sample selection, each set included only samples that originated from different projects and were classified into different types by ChIP-Atlas.

For samples considered to represent cell lines, the corresponding Cellosaurus terms were assigned and defined as the correct mappings. The final gold standard dataset was validated through manual inspection by two developmental biology experts and two bioinformatics experts, ensuring a high level of reliability.

Out of the selected samples, the number identified as representing cell lines was 183 for the ChIP-seq set and 139 for the ATAC-seq set. Among these, 17 and 12 samples, respectively, were confirmed to be cell lines but lacked corresponding terms in Cellosaurus. We recognize that such samples may include instances in which submitters have assigned unique names to cell lines.

Comparison of Existing Methods and LLM-Assisted Approaches Using the Gold Standard Dataset

Using the constructed gold standard dataset, we evaluated whether concept extraction using an LLM improved existing methods. Cell line names were extracted using the Llama 3.1 70B model, following the workflow illustrated in Fig. 1.

We compared the LLM-assisted pipeline with the MetaSRA pipeline using the metrics described in the Methods section (Table 4). The gold standard dataset included samples from both ChIP-seq and ATAC-seq experiments to evaluate the effect of gene names on the task, but the LLM did not mistakenly extract gene names as cell line names in any case, and no considerable differences were observed between the two types of samples (Supplementary Table 1). Conventional methods achieved high accuracy in mapping cell line samples to ontology terms by restricting the attribute names used. This conservative strategy also enabled a high probability of correctly identifying non-cell line samples. However, this approach came at the cost of cell line coverage, resulting in many actual cell line samples being left unmapped. In contrast, the LLM-assisted method achieved high coverage in ontology mapping for cell line samples without compromising accuracy by selecting the most appropriate strings from all available attributes. At the same time, the samples that remained unmapped to cell line terms were more likely to be genuinely non-cell line samples. This underscores the efficacy of LLM-based methods in enhancing the quality of automatic metadata curation.

Table 4. Evaluation of the cell line extraction task using conventional and proposed methods

Pipeline	Cell line accuracy	Cell line coverage	Non-cell line precision	Non-cell line recall
MetaSRA	0.903	0.721	0.782	0.937

LLM-assisted	0.923	0.930	0.940	0.934

Table 5 shows the categorization and quantification of the errors made by the LLM for samples when it failed to produce the correct output. Note that this categorization does not necessarily cover all possible errors that may occur in future executions. The most common error involved cases in which the input mentioned a cell line, but the sample represented a derivative of that cell line rather than the cell line itself. The LLM was likely to incorrectly identify these as cell lines. In other cases, the LLM overlooked cell line names in the input metadata. Among the eight samples where this failure occurred, six did not have attributes including either “cell line” or “cell type” in their keys. While the remaining two samples had a “cell type” attribute, the strings that should have been extracted were relatively short (“H1” and “JK1”).

Ontology mapping of the extracted strings resulted in multiple candidate Cellosaurus terms for 26 samples. The LLM was tasked with selecting the most likely cell line from the candidates (Prompt 2). The prompt instructed the LLM to withhold judgment when the information provided in the BioSample metadata was insufficient to narrow down the candidates.

Of the 26 samples, eight were judged incorrect for reasons other than “Selection failure” in Table 5. Among the remaining 18 samples, the LLM correctly selected the appropriate cell line for 11 samples and appropriately withheld judgment for one sample. In four cases, a single cell line was incorrectly selected when it should have withheld judgment due to insufficient information. In two cases, it incorrectly withheld judgment when it was expected to identify the appropriate cell line based on the BioSample descriptions. Taken together, for samples where a decision was feasible, the LLM achieved 11 correct answers out of 13.

These results suggest that an LLM can be effectively employed for tasks requiring the differentiation of identically named cell lines.

These findings suggest that while some challenges remain in disambiguating the sample context and ensuring comprehensive extraction, an LLM-assisted approach can substantially improve performance.

Table 5. Categorization of the errors made by the LLM.

Category	Description	#
Derivation	The sample was not the cell line itself but was derived from the cell line.	12
Overlook	The cell line name was overlooked by the LLM.	8
Non-canonical name	The cell line name was not canonical and did not correspond to any Cellosaurus terms.	8
Selection failure	The LLM failed to select the correct mapping from multiple candidates.	6
Wrong extraction	Extracted string did not represent the cell line name.	5
Ontology insufficiency	The term in Cellosaurus matching the string did not actually represent the cell line.	2
	Total	41

Evaluation of a Potential Application Extraction of Experimentally Altered Gene Names and Techniques

Based on the evaluation results of the cell line extraction task, we concluded that concept extraction using LLMs can be performed at a practical level for biological experimental factors. With this in mind, we aimed to enhance the utility of existing applications by applying similar methods to concepts not yet covered by manual curation.

We attempted to extract information about genes whose expression was experimentally modulated from the metadata of experimental samples collected by ChIP-Atlas. As described in the Methods section, we used a total of 3,723 samples, consisting of 1,752 ATAC-seq

samples and 1,971 ChIP-seq samples. Using Prompt 3 (as shown in the Methods section) for extraction, at least one gene was identified in 600 of the 3,723 samples. These results were manually evaluated for correctness, separately assessing the accuracy of the gene names and method names. We excluded samples for which a single correct answer was not clearly defined in the prompt, as these could be judged as either correct or incorrect, depending on different use cases. Examples included samples mentioning fusion genes, where it was unclear whether individual gene names within a fusion should be extracted separately or combined using notation, such as hyphens or a double colon ("::"). Other examples were samples with mutated genes. In some cases, the LLM output "mutation" as a method name, even when the input lacked this word, while in other cases, it extracted terms such as "K36M," exactly as described. Both were deemed reasonable in practice, but we excluded them from the evaluation because the prompt did not define which was correct.

Out of the 600 extractions, 579 cases were evaluable for both gene names and method names, and the accuracy rate was 0.803. When evaluated separately, the accuracy for gene names was 0.916, and the accuracy for method names was 0.847.

The extraction results included 459 unique gene names. Using the HGNC multi-symbol checker, 396 of these were mapped to one or more HGNC IDs. Among these, 32 were assigned multiple IDs and could not be uniquely resolved. Although gene symbols defined by HGNC are unique across all human genes, they are not always unique when synonyms are included. The information described in BioSample alone was typically insufficient to distinguish between them, representing a challenge for future work. In addition, 63 gene names could not be mapped to any corresponding ID. These cases included scenarios in which genes from non-human organisms, such as green fluorescent protein (GFP), had been introduced, as well as cases in which common names employing Greek letters not recognized by HGNC nomenclature were used.

Coverage was not evaluated in this study due to the absence of pre-existing manually curated results. However, the results of the accuracy evaluation demonstrate the potential for LLM-assisted extraction to considerably reduce the effort required by database users during sample searches.

Extraction results judged as incorrect often involved complex descriptions. Table 6 provides examples of such cases. These include, for example, a sample in which only the name of an inhibitor was mentioned, and additional information was required to determine the affected gene. Another example was a sample in which only the transduced gene carried an amino acid substitution mutation. These presented challenges, as describing them comprehensively requires defining a complex schema.

Designing prompts to account for every possible case is impractical. Instead, each application requires the user to find an appropriate balance of accuracy and coverage based on the specific needs of the situation.

Table 6. Examples of BioSample records with attributes that were difficult to describe with a simple schema

BioSample ID	Experiment Type	BioSample Attributes	Extracted Genes	Extracted Methods	Comments
SAMN03856375	ChIP-Seq	{"accession":"SAMN03856375","cell line":"K562","chip antibody":"anti-FLAG","chip antibody vendor":"Sigma-Aldrich","grna target":"globin HS2 enhancer","organism":"Homo sapiens","source_name":"Cultured K562 cells_dCas9_KRAB_HS2_CR10_FLAG","title":"dCas9_KRAB_HS2_CR10_FLAG_rep2","transduced gene":"dCas9-KRAB"}	dCas9-KRAB	transduction	dCas9-KRAB is indeed mentioned in the “transduced gene” attribute, but this is transduced to target the globin HS2 enhancer.
SAMN04226998	ChIP-Seq	{"accession":"SAMN04226998","cell line":"IMR90","cell type":"human diploid fibroblast","chip antibody":"mouse monoclonal H3K36me3 antibody, clone CMA333 (PMID: 20824077)","condition":"pApo; pro-apoptotic (overexpression of E1A/RasG12V)","genotype/variation":"overexpressing E1A/RasG12V","histone marks to be tested":"K36me3","organism":"Homo sapiens","source_name":"human diploid fibroblast","title":"Apo IMR90 H3K36 me3"}	E1A<br>RasG12V	overexpression<br>overexpression	E1A is actually a gene of Adenovirus. Without this knowledge, mapping to a gene ID is likely to fail.
SAMN06700885	ChIP-Seq	{"accession":"SAMN06700885","cell line":"SERPINE2 enhancer_KO#2","cell type":"colorectal cancer cell line","chip antibody":"Pol II (CST, catalog# 14958, lot# 1)","organism":"Homo sapiens","shrna":"PAF1 shRNA","source_name":"HCT116","title":"pol2.SERPINE2_enhancer_KO#2.shPAF1.rep1"}	SERPINE2<br>PAF1	knockout<br>knockdown	The term “KO” is mentioned, but only an enhancer of SERPINE2 is knocked out. The expression of SERPINE2 is considered to be affected, but classifying SERPINE2 as a knocked-out gene is incorrect.
SAMN08370440	ATAC-Seq	{"cell line":"G401", "passage":"ten-thirty", "source_name":"MRT cells", "title":"ATAC-seq OMOMYC rep3", "transfection":"OMOMYC"}	(null)	OMOMYC	“OMOMYC” is mentioned in the “transfection” attribute, but this is an inhibitor of MYC. The targeted gene name is not directly mentioned in the metadata.
SAMN0893	ATAC-Seq	{"cell line":"MOLM13", "cell	CBS79	knockout	“CBS79” means CTCF

7812		types":"Human-derived acute myeloid leukemia cells", "genotype/variation":"CBS79KO", "source_name":"MOLM13_CBS79KO_ATAC-seq", "title":"CBS79KO_1_ATAC-seq"}			Binding Site 7/9. To determine that this is not a gene name, advanced background knowledge is required.
SAMN10579999	ChIP-Seq	{"accession":"SAMN10579999","cell line":"22RV1","crispr clone":"no","foxa1 antibody":"CST","foxa1 genotype":"WT/WT + exo I176M","organism":"Homo sapiens","overexpression":"yes","source_name":"22RV1","target":"FOXA1","title":"22rv1-foxa1-i176m-v5-foxa1-cst-rep2"}	FOXA1	overexpression	Only exogenous FOXA1 has a mutation I176M. A complex schema is required to retain this information in the output.
SAMN14167723	ChIP-Seq	{"accession":"SAMN14167723","cell line":"Jurkat","chip antibody":"Flag","genotype/variation":"ZBTB1 KO expressing FLAG-ZBTB1 cDNA","organism":"Homo sapiens","source_name":"Jurkat cells","title":"ZBTB1 KO + ZBTB1 cDNA FLAG No Asparagine","treatment":"Asparagine deprivation"}	ZBTB1<br>ZBTB1	knockout<br>overexpression	Endogenous ZBTB1 is knocked out, and FLAG-tagged ZBTB1 is expressed. Classifying this as a ZBTB1 knocked-out gene can cause misunderstanding.
SAMN21208736	ATAC-Seq	{"cell line":"T265", "cell type":"MPNST", "source_name":"T265 cells", "title":"T265-SUZ12 no Dox ATAC rep2", "transduced with":"transduced with Dox-inducible SUZ12-ORF", "treatment":"untreated"}	SUZ12	overexpression	"transduced with Dox-inducible SUZ12-ORF" is mentioned, but the value of the "treatment" attribute is "untreated." SUZ12 was not considered overexpressed in this sample.

Discussion

Outcomes

In this study, we quantitatively confirmed the effectiveness of LLMs for extracting cell line names—a concept that has already been subject to manual curation—using samples covered by ChIP-Atlas. We further applied the same approach to the extraction of gene names that had been experimentally modulated. When searching ChIP-seq experimental data using simple string matching, the gene names retrieved often represented a mix of targets used in ChIP experiments and targets subjected to manipulations, such as knockouts. The current

version of ChIP-Atlas allows for the filtering of experiments only by the type of cells or tissues used. However, within the same classification, there can be samples in which the expressions of some genes have been experimentally modulated. By curating such information with LLMs, users could exclude samples involving KOs or KDs to reduce noise in their analyses and focus on more relevant results.

Although LLMs are expected to assist in correcting the low-quality metadata generated by humans, efforts to prevent the creation of such low-quality metadata in the first place remain essential. For example, NCBI has been working to improve the quality of submitted metadata by introducing additional constraints that metadata must meet upon submission to BioSample and by enhancing its documentation [22]. Tools such as CEDAR [23] are also available to assist in metadata creation. Data submitters should take advantage of these support systems and recognize that submitting data to public repositories is intended to enable data reuse. While data submitters should ensure that their metadata are properly described, we also understand that errors and mistakes can be published unintentionally. Our results improve the usability of published data that have these errors as part of their original submission.

Under the evaluation environment used in this study, the LLM could process approximately 400 samples per hour. The total number of epigenomics experiments included in ChIP-Atlas is approximately 430,000, which can be processed within a practical timeframe, enabling the benefits of LLM-based curation to be directly translated into greater user utility. Still, it should be noted that the total number of records in BioSample exceeds 40 million, and addressing this larger scale would require additional pre-processing or advancements in model performance.

Another key contribution of this research is demonstrating the utility of Llama 3, a locally deployable model. While many studies rely on commercial models such as GPT by OpenAI [24], our adoption of a local model ensures transparency and sustainability, avoiding

dependence on specific vendors. Additionally, for large-scale and continuous data processing, relying on paid services could raise sustainability concerns. Moreover, using a local model such as Llama 3 makes it feasible to apply similar methods to sensitive data, such as electronic health records, where privacy is paramount. Our approach is also more flexible than methods that depend on fixed schemas, such as those employed by the CEDAR group [17], allowing term extraction from arbitrary text rather than requiring adherence to predefined structures. As demonstrated by Cinquin's application to LLaMA, fine-tuning is one approach to improving the performance of LLMs on specific tasks, but considering the heterogeneity of BioSample records, constructing a training set that adequately captures the diversity of the records is challenging. From a practical standpoint, it is desirable to apply a general-purpose model without additional fine-tuning. Our results indicate that the newer Llama 3.1 model performs sufficiently well without fine-tuning, outperforming earlier efforts. Similarly, although Cinquin enhanced performance through prompts incorporating the chain-of-thought technique, our findings suggest that with Llama 3.1, the "think step by step" method alone yields satisfactory results, potentially eliminating the need to craft task-specific sets of questions tailored to each concept to be extracted.

Limitations

Despite the advances made with this research, several challenges remain unresolved:

1. **Complex metadata descriptions**. Experimental sample metadata can be intricate, making it difficult to represent some cases with the simple schema used in this study (as illustrated in the two examples below).
    - **Differentiated cell types**. When describing samples of cells differentiated from a specific cell line, ideally, both the original cell line and the differentiated cell type should be recorded.

- **Fusion proteins**. For gene name extraction, mapping to NCBI Gene IDs is complicated because NCBI Gene lacks entries for fusion genes. This necessitates using individual gene IDs and designing schemas that convey information about the fusion gene as a whole, not just its components.

While schema design and prompt engineering can partially address these issues, complete automation remains challenging.

2. **Limits of prompt engineering**. While improvements in model performance may yield better results for the same prompts, predicting the extent of these improvements is difficult.
3. **Computational constraints**. Processing the entirety of BioSample would require extensive computational resources, time, and energy. These constraints necessitate careful consideration of the practical scope and application of LLM-based approaches for each specific task.
4. **Lack of validation across broader conditions.** Although we conducted quantitative evaluations of the model performance, the samples analyzed were limited to specific experiment types (ChIP-seq and ATAC-seq) and a single species (human). While we anticipate that the proposed approach can be useful for other experiment types and organisms, we cannot guarantee comparable accuracy across all settings.

In light of these limitations, achieving fully comprehensive results with current LLMs may not be feasible for all tasks. Instead, it is essential to define appropriate use cases and balance expectations based on available resources and application needs.

Future Directions

The rapid advancement of LLMs holds considerable promise for tasks such as experimental metadata curation. As more powerful models become available, we anticipate further improvements in performance. As ChIP-Atlas’s manual curation results demonstrated

usefulness for this research, human curation remains valuable for providing near-complete curation and for evaluating the effectiveness of automated methods. Still, LLMs are poised to considerably reduce the workload of human curators.

This study represents an initial step in this direction, laying the groundwork for future applications and refinements. With continued development, LLM-based methods are expected to play a critical role in bridging the gap between large-scale metadata and efficient, accurate curation processes.

Abbreviations

ATAC-seq: Assay for transposase-accessible chromatin with sequencing

ChIP-seq: Chromatin immunoprecipitation followed by sequencing

DDBJ: DNA Databank of Japan

EBI: European Bioinformatics Institute

GEO: Gene Expression Omnibus

GFP: Green fluorescent protein

GPT: Generative pre-trained transformer

GPU: Graphics processing unit

HGNC: HUGO Gene Nomenclature Committee

KD: Knockdown

KO: Knockout

LLM: Large language model

NCBI: National Center for Biotechnology Information

NER: Named entity recognition

SRA: Sequence Read Archive

VRAM: Video Random Access Memory

Acknowledgments

We would like to thank Dr. Bernstein and colleagues, the developers of the original MetaSRA.

Computations were partially performed on the NIG supercomputer at the ROIS National Institute of Genetics.

Author Contributions

Shuya Ikeda (conceptualization, data curation, software, formal analysis, methodology, writing – original draft)

Zhaonan Zou (data curation, writing – review & editing)

Hidemasa Bono (writing – review & editing, project administration)

Yuki Moriya (methodology, writing – review & editing)

Shuichi Kawashima (writing – review & editing)

Toshiaki Katayama (writing – review & editing)

Shinya Oki (data curation, writing – review & editing)

Tazro Ohta (conceptualization, data curation, analysis, writing – review & editing, funding acquisition, project administration, supervision)

The use of AI tools in paper writing

The translation of the draft from Japanese to English was assisted by ChatGPT.

Funding

This work was supported by JST NBDC as part of the Development of fundamental technologies Project.

This work was supported by JSPS KAKENHI Grant Number 24K20889. This work was supported, in part, by ROIS-DS-JOINT (045RP2023, 039RP2024) to T. Ohta.

Competing Interests

The authors declare no competing interests.

Data Availability

The datasets used for the evaluation tasks are available at https://doi.org/10.5281/zenodo.14881142. This repository includes:

- A gold standard dataset for the cell line ontology mapping task
- The BioSample dataset used for the cell line ontology mapping task
- The results of the cell line ontology mapping of the test dataset using the LLM-assisted pipeline
- The results of the cell line ontology mapping of the test dataset by directly using the MetaSRA pipeline
- The BioSample dataset used for the gene name extraction task
- The results of the gene name extraction from the test dataset using the LLM-assisted pipeline

Availability of source code

Project name: bsllmner

Project home page: https://github.com/sh-ikeda/bsllmner

Operating system(s): Platform independent

Programming language: Python

Other requirements: None

License: MIT

Any restrictions to use by non-academics: none

Project name: MetaSRA

Project home page: https://github.com/sh-ikeda/MetaSRA-pipeline

Operating system(s): Platform independent

Programming language: Python

Other requirements: None

License: BSD-3-Clause

Any restrictions to use by non-academics: None

References

1. Katz K, Shutov O, Lapoint R, Kimelman M, Brister JR, O'Sullivan C. The Sequence Read Archive: a decade more of explosive growth. Nucleic Acids Res. 2022 Jan 7;50(D1):D387–90.
2. Clough E, Barrett T. The Gene Expression Omnibus database. Methods Mol Biol. 2016;1418:93–110.
3. Ziemann M, Kaspi A, El-Osta A. Digital expression explorer 2: a repository of uniformly processed RNA sequencing data. GigaScience. 2019 Apr 3;8(4):giz022.
4. Mahi NA, Najafabadi MF, Pilarczyk M, Kouril M, Medvedovic M. GREIN: An Interactive Web Platform for Re-analyzing GEO RNA-seq Data. Sci Rep. 2019 May 20;9(1):7580.

5. Zou Z, Ohta T, Oki S. ChIP-Atlas 3.0: a data-mining suite to explore chromosome architecture together with large-scale regulome data. Nucleic Acids Res. 2024 Jul 5;52(W1):W45–53.

6. Barrett T, Clark K, Gevorgyan R, Gorelenkov V, Gribov E, Karsch-Mizrachi I, et al. BioProject and BioSample databases at NCBI: facilitating capture and organization of metadata. Nucleic Acids Res. 2012 Jan 1;40(D1):D57–63.

7. Gonçalves RS, Musen MA. The variable quality of metadata about biological samples used in biomedical experiments. Sci Data. 2019 Feb 19;6(1):190021.

8. Mungall CJ, Torniai C, Gkoutos GV, Lewis SE, Haendel MA. Uberon, an integrative multi-species anatomy ontology. Genome Biol. 2012 Jan 31;13(1):R5.

9. Bard J, Rhee SY, Ashburner M. An ontology for cell types. Genome Biol. 2005;6(2):R21.

10. Schriml LM, Munro JB, Schor M, Olley D, McCracken C, Felix V, et al. The Human Disease Ontology 2022 update. Nucleic Acids Res. 2021 Nov 10;50(D1):D1255–61.

11. Bernstein MN, Doan A, Dewey CN. MetaSRA: normalized human sample-specific metadata for the Sequence Read Archive. Bioinformatics. 2017 Sep 15;33(18):2914–23.

12. Bairoch A. The Cellosaurus, a Cell-Line Knowledge Resource. J Biomol Tech. 2018 Jul;29(2):25–38.

13. Malone J, Holloway E, Adamusiak T, Kapushesky M, Zheng J, Kolesnikov N, et al. Modeling sample variables with an Experimental Factor Ontology. Bioinformatics. 2010 Apr 15;26(8):1112–8.

14. Klie A, Tsui BY, Mollah S, Skola D, Dow M, Hsu CN, et al. Increasing metadata coverage of SRA BioSample entries using deep learning–based named entity recognition. Database. 2021 Sep 29;2021:baab021.

15. Fang L, Chen Q, Wei CH, Lu Z, Wang K. Bioformer: an efficient transformer language model for biomedical text mining. 2023. https://arxiv.org/abs/2302.01588v1. Accessed 2 May 2025.

16. Dagdelen J, Dunn A, Lee S, Walker N, Rosen AS, Ceder G, et al. Structured information extraction from scientific text with large language models. Nat Commun. 2024 Feb 15;15(1):1418.

17. Sundaram SS, Solomon B, Khatri A, Laumas A, Khatri P, Musen MA. Use of a Structured Knowledge Base Enhances Metadata Curation by Large Language Models. 2024. http://arxiv.org/abs/2404.05893. Accessed 19 Jun 2024.

18. Cinquin O. ChIP-GPT: a managed large language model for robust data extraction from biomedical database records. Briefings in Bioinformatics. 2024 Mar; 25(2): bbad535

19. Ollama. https://ollama.com. Accessed 17 Dec 2024.

20. Introducing Llama 3.1: Our most capable models to date. Meta AI. https://ai.meta.com/blog/meta-llama-3-1. Accessed 17 Dec 2024.

21. Kojima T, Gu SS, Reid M, Matsuo Y, Iwasawa Y. Large Language Models are Zero-Shot Reasoners. 2023. http://arxiv.org/abs/2205.11916. Accessed 2 Dec 2024.

22. Upcoming Changes to NCBI's BioSample Database. NCBI Insights. 23 Oct 2024. https://ncbiinsights.ncbi.nlm.nih.gov/2024/10/23/changes-ncbis-biosample-database/. Accessed 23 Jan 2025.

23. Gonçalves RS, O'Connor MJ, Martínez-Romero M, Egyedi AL, Willrett D, Graybeal J, et al. The CEDAR Workbench: An Ontology-Assisted Environment for Authoring Metadata that Describe Scientific Experiments. Proc Int Semantic Web Conf. 2017 Oct;10588:103–10.

24. ChatGPT. https://chat.openai.com. Accessed 27 Dec 2023.

Click here to access/download
Supplementary Material
LLM_curated_BioSample_Supplementary.docx
