# Supplementary material for: Extraction of biological terms using large language models enhances the usability of metadata in the BioSample database
Source: Gigascience. 2025 Jun 23;14:giaf070. doi: 10.1093/gigascience/giaf070 (PMC12205978; doi:10.1093/gigascience/giaf070)
Supplement: giaf070_Authors_Response_To_Reviewer_Comments_original_submission [file giaf070_authors_response_to_reviewer_comments_original_submission.pdf]

Author's Response To Reviewer Comments

We deeply thank the reviewers for their positive comments and suggestions. Our responses follow each reviewer contribution in the text below.

Responses to Reviewer #1

> 1. The gold-standard dataset constructed for evaluation, though carefully validated by experts, was limited to 600 samples (300 ChIP-seq and 300 ATAC-seq). Such a limited scope may introduce selection bias or fail to capture the full variability present across the entire BioSample database (>40 million records). It is unclear how representative these samples are of real-world metadata submissions. Clearly demonstrate the representativeness of the sample selection or increase sample size to better represent BioSample's diversity.

Response:
We agree with the reviewer’s assessment of the natural limitation of sampling large populations. The BioSample database contains many idiosyncratic descriptions, such as those that have been provided by particular projects or data providers. Given the nature of the database, it would be challenging (if even possible) to select a dataset that could actually represent the entire BioSample database or even a large portion of it. In addition, we do not believe that increasing the size of the dataset used would dramatically change the conclusions of our study, despite the added resources and effort exerted. It is also nearly impossible to study the overall performance on the entire BioSample dataset, which is still growing.

ChIP-Atlas aggregates a comprehensive set of ChIP-seq and ATAC-seq datasets, and for our gold standard set, we randomly selected samples from different projects and cell types to mitigate potential bias. Based on the characteristics of the experimental procedure of these two sequencing techniques, it is reasonable to assume that the method’s performance would be similar with a DNA/RNA sequencing dataset. However, metagenomic samples are more diverse, and our approach may not be sufficient to capture the characteristics of those types of samples. We do acknowledge that our dataset did not represent the full diversity of BioSample. Thus, we have explicitly added this information to the limitations portion of the Discussion section.

> 2. The manuscript predominantly compares the proposed LLM-based approach to the MetaSRA pipeline. While MetaSRA is a relevant baseline, the omission of comparisons with other contemporary methods like ChIP-GPT, and Bioformer is a notable oversight. These tools represent significant advancements in the field and have demonstrated efficacy in tasks closely related to the study's objectives. A comprehensive evaluation against these methods or comparative discussions would provide a clearer understanding of the proposed approach's relative performance and contributions.
https://academic.oup.com/bib/article/25/2/bbad535/7600389
https://pmc.ncbi.nlm.nih.gov/articles/PMC10029052/

Response:
We acknowledge this oversight regarding recent work, such as ChIP-GPT and Bioformer. ChIP-GPT has fine-tuned the LLaMA 1.0 model and incorporates prompt-engineering techniques, such as chain-of-thought prompting and pre-summarization steps, to perform named entity recognition (NER). Bioformer is a lightweight BERT-based model that can perform NER of biological concepts and is characterized by its compactness without sacrificing accuracy. We recognize that both are important recent works in regard to how machine-learning methods can provide solutions to the challenges of handling biological text and metadata.

We believe that our work would be better characterized as the next phase of the work illustrated by these examples rather than as being in competition with them. A key difference between our work and these studies lies in ontology mapping. Rather than merely extracting concepts, our approach links them to standardized ontologies, enabling an ontology-driven search that leverages their rich semantic information.

We have updated the Introduction section to include a discussion of these prior works, and we have emphasized that our study directly addresses a research direction that these methods do not.

> 3. "LLM-assisted methods outperformed traditional approaches, achieving higher accuracy and coverage." While the study reports improved performance over MetaSRA, the absence of comparisons with other SOTA methods renders this assertion less robust. Without such comparative analyses, it's challenging to attribute the observed improvements solely to the proposed approach. Rephrasing claims to accurately reflect the scope of the comparisons made would strengthen clarity.

Response:
Further extending our response to the previous comment, to the best of our knowledge, no prior studies have performed ontology mapping on strings extracted using LLMs or similar methods. Therefore, we believe that comparing our method against algorithmic approaches, such as MetaSRA, is a valid and appropriate benchmark.

Moreover, one of the key contributions of our study is to demonstrate that recent general-purpose language models without domain-specific fine-tuning have made concept extraction considerably more accessible and accurate. We have revised the manuscript to clarify this point, emphasizing that the reported improvements highlight both an advancement in model capabilities and the novelty of incorporating ontology mapping.

> 4. Despite high accuracy, complex cases (fusion proteins, inhibitors mentioned indirectly, ambiguous terminology) were recognized as difficult, yet were excluded from primary accuracy evaluations. By excluding these ambiguous cases from performance metrics, the accuracy results might be artificially improved. Provide additional metrics that include these complex or ambiguous cases, clearly quantifying performance drops. This would offer more realistic insights into real-world applicability.

Response:
We excluded certain cases from the evaluation, not because of their complexity but because of the ambiguous nature of the problem. We found that the output of the LLM could be judged as correct or incorrect, depending on certain use cases. We did not anticipate these cases prior to our experiment; our prompt would have required a change to fairly evaluate performance with their inclusion. We have revised the manuscript to emphasize this point more clearly.

Given the context of the gene name extraction cases, which was a real-world application development trial, the primary purpose of reporting the accuracy was not to assert the superiority of our method but rather to provide a practical reference for how often incorrect outputs occurred under the given conditions and what developers and users could potentially expect. Including such context-sensitive cases in a single aggregated metric could misrepresent the true utility of the method, which is ultimately dependent on the user’s needs.

> 5. The error categorization provided (derivation issues, overlooked terms, selection failures, etc.) is helpful, but somewhat superficial. The deeper root causes—such as the LLM's lack of biological context knowledge, tokenization errors, or prompt ambiguity—were not thoroughly explored or explained. Discuss or perform deeper qualitative analysis on specific error instances, highlighting precisely why the LLM made incorrect decisions (e.g., lack of biological understanding, misinterpretation of abbreviations, limitations of prompt wording).

Response:
It is inherently difficult to identify precisely the root causes of LLM errors, as the decision-making processes of the models are black boxes. While certain tasks that were not possible with LLaMA 1 have become feasible with LLaMA 3, the only plausible inference from the user’s standpoint is that the overall model performance has improved. We cannot determine whether a model truly possesses “biological context” knowledge, nor can we exhaustively explore all possible prompt variations, as prompt optimization is an

open-ended process. Given these limitations, our focus was on whether the method performed sufficiently well for practical applications rather than on speculative analysis of the model internals.

> 6. Temperature settings were fixed at zero for deterministic outputs. While deterministic settings are valuable for reproducibility, exploring or reporting the effect of temperature variations on accuracy and robustness would have strengthened this methodological choice significantly.

Response:
We agree with the comment that it would be interesting to explore how temperature settings can affect overall performance. However, as stated in the manuscript, we aimed to develop a practical method that we could implement in real-world use cases to improve the usability of bioinformatics resources, such as ChIP-Atlas. In this context, as the reviewer agreed, temperature tuning would affect reproducibility, so we could not use it as part of the database development.

We believe that the reproducibility and transparency of LLM methods are factors that cannot be ignored by application developers in bioinformatics. Exploring the parameters and prompts that improve performance will likely not be a top priority for the time being, as the current nature of LLMs is such that their overall performance will improve as new models become available.

> 7. The authors have not sufficiently explored or justified their prompt engineering choices which are critical for reproducibility and optimization. I recommend providing additional experiments or discussions on alternative prompting strategies tested, including prompt variants that failed and reasons why particular prompts were selected.

Response:
We agree that exploring improvements through techniques such as chain-of-thought prompting, as done in ChIP-GPT, is certainly a viable approach. However, we observed that our simple prompt formulation had already achieved a performance comparable to that of ChIP-GPT. This suggests that performance improvements can be achieved through model refinement. We believe that we have demonstrated the practical value of the fact that general-purpose models perform very well with simple prompts, reducing the need for intensive prompt engineering. Within the context of the rapid pace of model improvement, we believe that the contribution of this research is the demonstration of strong performance with minimal prompt design.

Responses to Reviewer #2

> There are only a few minor issues in language usage and grammar that require attention. For example, there is a small typo in the description of gene overexpression ("achieved by trasfection of a plasmid…" on page 19) - "trasfection" should be "transfection" (unless this typo was carried over from the original prompt). Another example is the sentence "the outcomes of this study can handle these errors to rescue the affected published data for further use," which is a bit awkward in phrasing - perhaps reword to clarify that the methods developed can help correct metadata errors from submitted data.

Response:
We noticed this typographical error after the evaluation, but the model was robust enough not to be affected by it. The results were still good; thus, we did not feel the need to perform the experiment again with a correction to the prompt. The prompt was reported accurately.

The manuscript was proofread before the initial submission and after our revision. We thank the reviewer for pointing out the text that still requires improvement. We have updated the sentence to clarify our original intent.

> Additional comments and suggestions: Beyond the points above, I have a few minor suggestions to

further strengthen the manuscript. First, it would be helpful if the authors could clarify in the Methods how the manual evaluation of gene name extraction was performed—for example, whether multiple curators independently reviewed the outputs or if any consensus procedure was employed to resolve ambiguous cases. Providing this detail would add transparency to the accuracy figures reported, although the existing explanation about handling ambiguous cases (e.g., fusion genes) is already helpful.

Response:
We have added details to the Methods section describing the manual curation process for gene name extraction, as follows:
"An initial assessment was performed by one curator, followed by a review by a more experienced curator. For complex cases, the final judgment was made through discussion among four curators."

> Second, given the manuscript's emphasis on a zero-shot LLM approach, it would be beneficial for the authors to briefly discuss whether alternative strategies, such as fine-tuning smaller language models, were considered. This would more clearly position the study within the broader landscape of metadata curation techniques.

Response:
Designing a fine-tuning dataset that adequately covers the diversity of BioSample metadata would be highly labor-intensive and difficult to use for generalization. From a practical standpoint, being able to achieve strong performance without fine-tuning—such as using zero-shot or few-shot prompting—is more desirable for real-world applications. Our results demonstrate that this is indeed feasible, and we have added a brief commentary on this point to the Discussion section to better position our approach within the broader landscape of metadata curation strategies.

> Third, the authors describe the use of the locally deployed Llama 3.1 model and emphasize its advantages regarding data privacy and scalability. Since these benefits are significant for practical adoption, it would further strengthen the manuscript if the authors explicitly highlight practical considerations, such as specific hardware requirements (in addition to the graphics card usage already included) and runtime performance benchmarks.

Response:
In the Methods section, we have added information about the required GPU memory size for running the Llama 3.1 model. Regarding runtime performance benchmarks, we considered this outside the scope of the current study, as there were no task-specific factors that would noticeably impact execution speed. However, we have already provided a general note on runtime performance in our Discussion section based on the specific tasks and computing environment used in the study.

> Finally, as mentioned earlier, the authors mention in Supplementary Table 1 that "no significant differences were observed" between ChIP-seq and ATAC-seq samples. If the term "significant" here is meant to indicate statistical significance, please include details of the specific statistical test and associated values (e.g., test statistics and p-values) that substantiate this conclusion. If no formal statistical testing was performed, it would be more appropriate to rephrase this statement to indicate a qualitative observation rather than imply statistical testing. These points are relatively minor and do not indicate fundamental issues with the manuscript.

Response:
As correctly pointed out by the reviewer, the statement in Supplementary Table 1 did not refer to statistical significance. We have rephrased the wording to clarify that this was a qualitative observation rather than the result of formal statistical inference testing.

Editorial Manager®

Cookie Preference Center

We use cookies which are necessary to make our site work. We may also use additional cookies to analyze, improve and personalize our content and your digital experience. For more information, see our Cookie Policy.

You may choose not to allow some types of cookies. However, blocking some types may impact your experience of our site and the services we are able to offer. See the different category headings below to find out more or change your settings.

Allow all

Manage Consent Preferences

Strictly Necessary Cookies

Always active

These cookies are necessary for the website to function and cannot be switched off in our systems. They are usually only set in response to actions made by you which amount to a request for services, such as setting your privacy preferences, logging in or filling in forms. You can set your browser to block or alert you about these cookies, but some parts of the site will not then work. These cookies do not store any personally identifiable information.

Functional Cookies

These cookies enable the website to provide enhanced functionality and personalisation. They may be set by us or by third party providers whose services we have added to our pages. If you do not allow these cookies then some or all of these services may not function properly.

Performance Cookies

These cookies allow us to count visits and traffic sources so we can measure and improve the performance of our site. They help us to know which pages are the most and least popular and see how visitors move around the site.

Targeting Cookies

These cookies may be set through our site by our advertising partners. They may be used by those companies to build a profile of your interests and show you relevant adverts on other sites. If you do not allow these cookies, you will experience less targeted advertising.

Cookie List

Clear

Apply Cancel
Consent Leg.Interest

Confirm my choices

Powered by onetrust
