# Supplementary material for: Extraction of biological terms using large language models enhances the usability of metadata in the BioSample database
Source: Gigascience. 2025 Jun 23;14:giaf070. doi: 10.1093/gigascience/giaf070 (PMC12205978; doi:10.1093/gigascience/giaf070)
Supplement: giaf070_Reviewer_1_Report_Original_Submission — Sajib Acharjee Dip -- 4/5/2025 [file giaf070_reviewer_1_report_original_submission.pdf]

Reviewer Report

Title: Extraction of biological terms using large language models enhances the usability of metadata in the BioSample database

Version: Original Submission **Date:** 4/5/2025

Reviewer name: Sajib Acharjee Dip

Reviewer Comments to Author:

1. The gold-standard dataset constructed for evaluation, though carefully validated by experts, was limited to 600 samples (300 ChIP-seq and 300 ATAC-seq). Such a limited scope may introduce selection bias or fail to capture the full variability present across the entire BioSample database (>40 million records). It is unclear how representative these samples are of real-world metadata submissions.Clearly demonstrate the representativeness of the sample selection or increase sample size to better represent BioSample's diversity.
2. The manuscript predominantly compares the proposed LLM-based approach to the MetaSRA pipeline. While MetaSRA is a relevant baseline, the omission of comparisons with other contemporary methods like ChIP-GPT, and Bioformer is a notable oversight. These tools represent significant advancements in the field and have demonstrated efficacy in tasks closely related to the study's objectives. A comprehensive evaluation against these methods or comparative discussions would provide a clearer understanding of the proposed approach's relative performance and contributions.
https://academic.oup.com/bib/article/25/2/bbad535/7600389
https://pmc.ncbi.nlm.nih.gov/articles/PMC10029052/
3. "LLM-assisted methods outperformed traditional approaches, achieving higher accuracy and coverage." While the study reports improved performance over MetaSRA, the absence of comparisons with other SOTA methods renders this assertion less robust. Without such comparative analyses, it's challenging to attribute the observed improvements solely to the proposed approach.â€‹ Rephrasing claims to accurately reflect the scope of the comparisons made would strengthen clarity.
4. Despite high accuracy, complex cases (fusion proteins, inhibitors mentioned indirectly, ambiguous terminology) were recognized as difficult, yet were excluded from primary accuracy evaluations. By excluding these ambiguous cases from performance metrics, the accuracy results might be artificially improved. Provide additional metrics that include these complex or ambiguous cases, clearly quantifying performance drops. This would offer more realistic insights into real-world applicability.
5. The error categorization provided (derivation issues, overlooked terms, selection failures, etc.) is helpful, but somewhat superficial. The deeper root causesâ€"such as the LLM's lack of biological context knowledge, tokenization errors, or prompt ambiguityâ€"were not thoroughly explored or explained. Discuss or perform deeper qualitative analysis on specific error instances, highlighting precisely why the LLM made incorrect decisions (e.g., lack of biological understanding, misinterpretation of abbreviations, limitations of prompt wording).
6. Temperature settings were fixed at zero for deterministic outputs. While deterministic settings are valuable for reproducibility, exploring or reporting the effect of temperature variations on accuracy and

robustness would have strengthened this methodological choice significantly.
7. The authors have not sufficiently explored or justified their prompt engineering choices which are critical for reproducibility and optimization. I recommend providing additional experiments or discussions on alternative prompting strategies tested, including prompt variants that failed and reasons why particular prompts were selected.

Methods

Are the methods appropriate to the aims of the study, are they well described, and are necessary controls included? Choose an item.

Conclusions

Are the conclusions adequately supported by the data shown? Choose an item.

Reporting Standards

Does the manuscript adhere to the journal's guidelines on minimum standards of reporting? Choose an item.

Choose an item.

Statistics

Are you able to assess all statistics in the manuscript, including the appropriateness of statistical tests used? Choose an item.

Quality of Written English

Please indicate the quality of language in the manuscript: Choose an item.

Declaration of Competing Interests

Please complete a declaration of competing interests, considering the following questions:

- Have you in the past five years received reimbursements, fees, funding, or salary from an organisation that may in any way gain or lose financially from the publication of this manuscript, either now or in the future?
- Do you hold any stocks or shares in an organisation that may in any way gain or lose financially from the publication of this manuscript, either now or in the future?
- Do you hold or are you currently applying for any patents relating to the content of the manuscript?
- Have you received reimbursements, fees, funding, or salary from an organization that holds or has applied for patents relating to the content of the manuscript?

- Do you have any other financial competing interests?
- Do you have any non-financial competing interests in relation to this paper?

If you can answer no to all of the above, write 'I declare that I have no competing interests' below. If your reply is yes to any, please give details below.

I declare that I have no competing interests.

I agree to the open peer review policy of the journal. I understand that my name will be included on my report to the authors and, if the manuscript is accepted for publication, my named report including any attachments I upload will be posted on the website along with the authors' responses. I agree for my report to be made available under an Open Access Creative Commons CC-BY license (http://creativecommons.org/licenses/by/4.0/). I understand that any comments which I do not wish to be included in my named report can be included as confidential comments to the editors, which will not be published.

Choose an item.

To further support our reviewers, we have joined with Publons, where you can gain additional credit to further highlight your hard work (see: https://publons.com/journal/530/gigascience). On publication of this paper, your review will be automatically added to Publons, you can then choose whether or not to claim your Publons credit. I understand this statement.

Yes Choose an item.
