# Supplementary material for: Extraction of biological terms using large language models enhances the usability of metadata in the BioSample database
Source: Gigascience. 2025 Jun 23;14:giaf070. doi: 10.1093/gigascience/giaf070 (PMC12205978; doi:10.1093/gigascience/giaf070)
Supplement: giaf070_Reviewer_2_Report_Original_Submission — Christopher Tabone, Ph.D. -- 4/8/2025 [file giaf070_reviewer_2_report_original_submission.pdf]

Reviewer Report

Title: Extraction of biological terms using large language models enhances the usability of metadata in the BioSample database

Version: Original Submission **Date:** 4/8/2025

Reviewer name: Christopher Tabone, Ph.D.

Reviewer Comments to Author:

This manuscript evaluates the use of large language models (LLMs) to improve the consistency and usefulness of BioSample metadata. The authors focus on extracting specific biological terms from free-text sample descriptions: first, identifying cell line names (using a curated gold-standard for evaluation), and second, identifying experimentally modulated gene names (in a scenario without prior manual curation). An open-source 70B LLM (Llama 3.1) was used and its performance was compared against a conventional ontology-mapping pipeline (MetaSRA). Overall, the study is well-motivated - addressing the challenge of heterogeneous metadata - and the approach is generally sound and well documented. Below, I address specific aspects of the work in detail:

Methodological Appropriateness and Controls: The methods are appropriate to the study's aims and are described with detail. The two-part evaluation (cell line extraction and gene name extraction without prior curation) aligns well with the goal of demonstrating LLM utility in metadata curation. The authors took care to construct a gold-standard dataset for cell line extraction by leveraging ChIP-Atlas's manually curated sample annotations. This approach avoids starting from scratch and ensures the evaluation is grounded in experimental metadata. The sample selection strategy is well justified: using equal numbers of ChIP-seq and ATAC-seq samples to control for the presence/absence of protein names (a potential confounder for detecting cell lines), avoiding duplicate projects and identical terms, and restricting to human samples to leverage the Cellosaurus ontology. These controls strengthen the evaluation by preventing bias (e.g. one project dominating results or trivial cases duplicating answers). The LLM pipeline is clearly outlined (Figure 2) - the model is prompted with BioSample attributes to extract a representative cell line term. Importantly, the authors compare this LLM-assisted pipeline against an existing rule-based method (the MetaSRA ontology mapping pipeline). This serves as an essential control/baseline to quantify the improvement gained by using an LLM. For the second task (extracting modulated gene names), where no curated baseline exists, the authors sample thousands of BioSample entries and perform manual evaluation of the LLM's outputs. While manual checking is necessary here, the manuscript could clarify the evaluation procedure (e.g. how many evaluators or what criteria were used) to assure readers of consistency. Overall, the experimental design is solid. The necessary details (model used, prompt design, parameter settings like temperature=0 for reproducibility) are all provided, and the authors have made their code publicly available, which aids reproducibility. The methodology is transparent and should allow others to replicate or build upon the work.

Support for Conclusions by Data: The conclusions are, for the most part, well supported by the data presented. In the cell line extraction task, the LLM-based method clearly outperforms the traditional MetaSRA pipeline in both accuracy and coverage (Table 4). For example, the LLM pipeline achieved

substantially higher coverage (93.0% vs 72.1% for MetaSRA) without sacrificing accuracy (~92.3% vs 90.3%), and it also showed improved precision in identifying non-cell line samples. These results validate the authors' claim that LLMs can more flexibly and comprehensively interpret metadata, mapping many more actual cell line samples to ontology terms while maintaining low false-positive rates. The data support the conclusion that the LLM approach enhances metadata findability (since far more samples get correctly annotated) and does so with high reliability. The authors appropriately note that the conventional method's conservative strategy yields high precision at the cost of leaving many samples unmapped, whereas the LLM can confidently map a greater portion of samples. This finding is well substantiated by the numbers and the error analysis in Table 5 (which categorizes the few failure cases of the LLM, such as confusion with derivative cell lines or missing a cell line when certain keywords were absent). In the gene name extraction task, the authors report that the LLM identified at least one gene in 600 out of 3,723 tested samples, with an overall accuracy of ~80.3% for those outputs (about 91.6% accuracy on gene names themselves, and 84.7% on the associated modulation method). This demonstrates that the LLM can successfully parse complex descriptions to find gene perturbations in a majority of cases. While there is no baseline for direct comparison here, these results are consistent with the idea that LLMs can extend curation to new information types not yet curated (in this case, finding manipulated genes where an ontology or curated list didn't exist). The authors' conclusions about the utility of this - for example, that it could allow users to filter out experiments with gene knockouts/knockdowns to avoid confounding effects - are reasonable extrapolations from the data. The discussion correctly notes that coverage for this gene task wasn't evaluated (since no gold standard exists) and acknowledges that some fraction of relevant cases might be missed. All major conclusions (LLM outperforms rule-based methods; LLM extraction of new metadata is feasible and useful) are backed by the evidence provided. The authors also contextualize their findings by noting limitations and practical considerations (e.g. the processing throughput of ~400 samples/hour and the challenge of scaling to 40 million records). This adds credibility to their interpretation that LLM-based curation will need further resources or model improvements to handle the entire database. In summary, the data presented are analyzed in depth (with relevant tables, figures, and a breakdown of error types), and they support the paper's conclusions well. I have no concerns that the authors are overstating their results.

Language Clarity and Quality: The manuscript is written in generally clear and professional English. The authors note that they translated the draft from Japanese with assistance from ChatGPT, and the result is readable and scientifically appropriate. The overall clarity is good - important terms are defined, and the narrative flows logically from the motivation to methods, results, and discussion. I did not encounter ambiguities that impede understanding of the science. There are only a few minor issues in language usage and grammar that require attention. For example, there is a small typo in the description of gene overexpression ("achieved by trasfection of a plasmid…" on page 19) - "trasfection" should be "transfection" (unless this typo was carried over from the original prompt). Another example is the sentence "the outcomes of this study can handle these errors to rescue the affected published data for further use," which is a bit awkward in phrasing - perhaps reword to clarify that the methods developed can help correct metadata errors from submitted data. These are relatively minor edits; the manuscript does not require heavy language revision, just light editing for a few misspellings and stylistic "smoothing".

The structure of the paper is appropriate, with a clear Introduction and well-labeled sections (Methods, Results/Discussion, Limitations, etc.). Data presentation is also clear: figures and tables are easy to interpret, and captions are explanatory. For example, the flowchart in Figure 2 and the definitions in Figure 3 clearly help in the understanding of the pipeline and metrics. In summary, with minor editorial changes, the quality of language and presentation will be suitable for publication.
Statistical Analysis and Data Presentation: I am able to assess all the statistics and quantitative analyses in the manuscript, and they appear appropriate. The study primarily uses descriptive performance metrics (accuracy, coverage, precision, recall) to evaluate the extraction tasks - these are standard and well defined (the text and Figure 3 provide clear definitions of each metric in the context of the task). The comparisons between the LLM pipeline and the MetaSRA pipeline are straightforward to interpret. The authors did not perform complex statistical tests (e.g., no p-values are reported), which can be justified given that the magnitude and consistency of the improvements are evident and the evaluation emphasizes practical performance metrics rather than hypothesis testing. However, the manuscript states in Supplementary Table 1 that "no significant differences were observed" between ChIP-seq and ATAC-seq subsets. If the authors intend "significant" to indicate statistical significance, it would be necessary to include the specific statistical test used along with associated test statistics and p-values to substantiate this claim. If no formal statistical testing was conducted, it would be more accurate and clearer to rephrase this as a qualitative observation rather than implying formal statistical support. All underlying data needed to interpret the results are provided either in the main figures/tables or supplementary material. The presentation of results is clear and transparent: Table 4 quantitatively summarizes the performance of each pipeline, and Table 5 qualitatively categorizes the errors made by the LLM. I have no other concerns about the appropriateness of statistical methods used - the evaluation metrics are suitable for information extraction tasks, and the sample sizes (600 samples for the cell line task, and thousands scanned for the gene task) are adequate to support the conclusions. In terms of data transparency, the manuscript indicates that outputs and code are available (with a GitHub repository provided), which will allow others to reproduce the analysis.
Additional comments and suggestions: Beyond the points above, I have a few minor suggestions to further strengthen the manuscript. First, it would be helpful if the authors could clarify in the Methods how the manual evaluation of gene name extraction was performedâ€”for example, whether multiple curators independently reviewed the outputs or if any consensus procedure was employed to resolve ambiguous cases. Providing this detail would add transparency to the accuracy figures reported, although the existing explanation about handling ambiguous cases (e.g., fusion genes) is already helpful. Second, given the manuscript's emphasis on a zero-shot LLM approach, it would be beneficial for the authors to briefly discuss whether alternative strategies, such as fine-tuning smaller language models, were considered. This would more clearly position the study within the broader landscape of metadata curation techniques. Third, the authors describe the use of the locally deployed Llama 3.1 model and emphasize its advantages regarding data privacy and scalability. Since these benefits are significant for practical adoption, it would further strengthen the manuscript if the authors explicitly highlight practical considerations, such as specific hardware requirements (in addition to the graphics card usage already included) and runtime performance benchmarks. Finally, as mentioned earlier, the authors mention in Supplementary Table 1 that "no significant differences were observed" between ChIP-seq and ATAC-seq samples. If the term "significant" here is meant to indicate statistical significance, please include details

of the specific statistical test and associated values (e.g., test statistics and p-values) that substantiate this conclusion. If no formal statistical testing was performed, it would be more appropriate to rephrase this statement to indicate a qualitative observation rather than imply statistical testing. These points are relatively minor and do not indicate fundamental issues with the manuscript.
Recommendation: In summary, this is a strong manuscript that addresses a pertinent problem in biological data management using modern LLM tools. The methods are sound and well controlled, the results are convincing, and the authors have been appropriately cautious and thorough in their analysis. I recommend minor revisions for this manuscript. The revisions needed are primarily editorial (minor language fixes and clarifications), with one note about statistics, and do not require additional experiments. With those addressed, the work should be suitable for publication in GigaScience.

Methods

Are the methods appropriate to the aims of the study, are they well described, and are necessary controls included? Choose an item.

Conclusions

Are the conclusions adequately supported by the data shown? Choose an item.

Reporting Standards

Does the manuscript adhere to the journal's guidelines on minimum standards of reporting? Choose an item.

Choose an item.

Statistics

Are you able to assess all statistics in the manuscript, including the appropriateness of statistical tests used? Choose an item.

Quality of Written English

Please indicate the quality of language in the manuscript: Choose an item.

Declaration of Competing Interests

Please complete a declaration of competing interests, considering the following questions:

- Have you in the past five years received reimbursements, fees, funding, or salary from an organisation that may in any way gain or lose financially from the publication of this manuscript, either now or in the future?
- Do you hold any stocks or shares in an organisation that may in any way gain or lose financially from the publication of this manuscript, either now or in the future?

- Do you hold or are you currently applying for any patents relating to the content of the manuscript?
- Have you received reimbursements, fees, funding, or salary from an organization that holds or has applied for patents relating to the content of the manuscript?
- Do you have any other financial competing interests?
- Do you have any non-financial competing interests in relation to this paper?

If you can answer no to all of the above, write 'I declare that I have no competing interests' below. If your reply is yes to any, please give details below.

I declare that I have no competing interests.

I agree to the open peer review policy of the journal. I understand that my name will be included on my report to the authors and, if the manuscript is accepted for publication, my named report including any attachments I upload will be posted on the website along with the authors' responses. I agree for my report to be made available under an Open Access Creative Commons CC-BY license (http://creativecommons.org/licenses/by/4.0/). I understand that any comments which I do not wish to be included in my named report can be included as confidential comments to the editors, which will not be published.

Choose an item.

To further support our reviewers, we have joined with Publons, where you can gain additional credit to further highlight your hard work (see: https://publons.com/journal/530/gigascience). On publication of this paper, your review will be automatically added to Publons, you can then choose whether or not to claim your Publons credit. I understand this statement.

Yes Choose an item.
